# Identifying PLAUR as a Pivotal Gene of Tumor Microenvironment and Regulating Mesenchymal Phenotype of Glioblastoma

**DOI:** 10.3390/cancers16040840

**Published:** 2024-02-19

**Authors:** Zaixiang Fu, Zihang Chen, Jingya Ye, Jianxiong Ji, Weifang Ni, Weibo Lin, Haopu Lin, Liquan Lu, Ganggui Zhu, Qin Xie, Feng Yan, Gao Chen, Fuyi Liu

**Affiliations:** 1 Department of Neurosurgery, The Second Affiliated Hospital of Zhejiang University School of Medicine, Hangzhou 310000, China; 22018424@zju.edu.cn (Z.F.); 12118478@zju.edu.cn (Z.C.); yjy300414@163.com (J.Y.); jijx@zju.edu.cn (J.J.); minendt@163.com (W.N.); 22218570@zju.edu.cn (W.L.); 22218574@zju.edu.cn (H.L.); 22218525@zju.edu.cn (L.L.); 18167167723@163.com (Q.X.); fengyanzju@zju.edu.cn (F.Y.); 2 Key Laboratory of Precise Treatment and Clinical Translational Research of Neurological Diseases, Hangzhou 310000, China; 3 Department of Lung Transplantation, The Second Affiliated Hospital of Zhejiang University School of Medicine, Hangzhou 310052, China; 22018428@zju.edu.cn

**Keywords:** glioblastoma, PLAUR, tumor microenvironment, mesenchymal phenotype, single-cell RNA sequencing

## Abstract

**Simple Summary:**

The mesenchymal (MES) phenotype of glioblastoma (GBM) confers resistance to various therapeutic strategies and results from both tumor-intrinsic genetic alterations and tumor-extrinsic microenvironmental factors. In this study, we sought to identify and validate the key genes that regulate the MES phenotype of glioma via both mechanisms. By integrating bulk tumor data (using the TCGA and CGGA databases) and single-cell sequencing data of GBM, we revealed that the plasminogen activator, urokinase receptor (PLAUR) is the hub gene of the protumor microenvironment, encompassing hypoxia and immunosuppression, and we elucidated its role in driving the MES transition through both tumor-intrinsic function and cell-to-cell interaction. Our findings indicate that PLAUR may be a potential target for GBM treatment, as our research elucidated its functions through a comprehensive study.

**Abstract:**

The mesenchymal (MES) phenotype of glioblastoma (GBM) is the most aggressive and therapy-resistant subtype of GBM. The MES phenotype transition during tumor progression results from both tumor-intrinsic genetic alterations and tumor-extrinsic microenvironmental factors. In this study, we sought to identify genes that can modulate the MES phenotype via both mechanisms. By integrating weighted gene co-expression network analysis (WGCNA) and the differential expression analysis of hypoxia-immunosuppression-related genes, we identified the plasminogen activator, urokinase receptor (PLAUR) as the hub gene. Functional enrichment analysis and GSVA analysis demonstrated that PLAUR was associated with the MES phenotype of glioma and the hypoxia-immunosuppression-related microenvironmental components. Single-cell sequencing analysis revealed that PLAUR mediated the ligand–receptor interaction between tumor-associated macrophages (TAMs) and glioma cells. Functional experiments in vitro with cell lines or primary glioma cells and xenograft models using BALB/c nude mice confirmed the role of PLAUR in promoting the MES phenotype of GBM. Our findings indicate that PLAUR regulates both glioma cells and tumor cell-extrinsic factors that favor the MES phenotype and suggest that PLAUR might be a potential target for GBM therapy.

## 1. Introduction

Glioblastoma (GBM) is the most common and aggressive type of primary brain tumor in adults and results in poor patient outcomes. Despite the use of multimodal therapies, including surgery, radiotherapy, and temozolomide (TMZ) chemotherapy, the median survival of GBM patients is only about 15 months due to the high risk of recurrence and progression [1,2]. Numerous studies have revealed that heterogeneity is one of the most important contributors of therapy resistance, and increasing intratumoral heterogeneity is considered as a robust indicator of a poor prognosis [3,4,5,6]. Briefly, heterogenous glioma subclones with distinct molecular characteristics and complex and variable tumor microenvironment components are two major factors contributing to the heterogeneity of GBMs [7,8]. To better understand the heterogeneity of GBMs and to identify effective treatment strategies, GBMs have been classified into different molecular subtypes based on their transcriptional profiles and mutation events. These subtypes include classical (TCGA-CL), mesenchymal (TCGA-MES), and proneural (TCGA-PN) [9]. The MES subtype is the most malignant, characterized by high radioresistance and the poorest prognosis for patients [10,11]. Due to intratumoral heterogeneity, different subtypes can coexist in different regions of the same tumor specimen. Thus, single-cell sequencing has been used to further investigate the heterogeneity of GBMs at the cellular level [12]. GBM cells exhibit high plasticity and variability in their molecular expression states, which can be categorized into four distinct cellular states: astrocyte-like (AC-like), oligodendrocyte progenitor-like (OPC-like), neural progenitor-like (NPC-like), and mesenchymal-like (MES-like) [13]. The MES-like state is similar to the TCGA-MES subtype, with a high frequency of NF1 alterations and increased immune infiltration [9]. Therefore, the MES phenotype is an essential concept for inter- and intratumor heterogeneity, and it may be a promising target in addressing GBM recurrence and progression.

The acquisition of the MES phenotype is a common phenomenon in the progression of GBM. In tumor recurrence, it can manifest as a characteristic PN-to-MES transition (PMT) and acquire resistance to antiangiogenic therapy and radiotherapy [11,14]. According to an integrating study on the GBM progression trajectory from the Glioma Longitudinal Analysis Consortium (GLASS), the phenotypic transition of GBM in the context of recurrence depends on changes in the cell-intrinsic genome, physical structures, and microenvironmental interactions [15]. Previous studies have clarified the contribution of cell-intrinsic gene alterations to the MES phenotype, such as the activation of the nuclear factor κb pathway regulated by the molecule GRP78 [16,17]. Recently, single-cell and spatial omics technologies have highlighted the importance of physical characteristics and immune components in the tumor microenvironment, in addition to the cell-intrinsic factors of glioma cells [18].

Hypoxia is a prominent physical feature of the tumor microenvironment that results from rapid tumor growth and abnormal vascularization [19]. GBM often exhibits severe hypoxia (as low as about 1% oxygen), which enhances the invasiveness and radioresistance of glioma and other MES-related phenotypes, as well as the genomic instability of tumor cells [20]. Moreover, hypoxia is associated with immune cell infiltration and suppressive microenvironment formation. Hypoxia regulates tumor cells to secrete cytokines, exosomes, and other extracellular mediators, which facilitate the recruitment of immune cells (especially macrophages) to the tumor core and modulate their function in the hypoxic milieu, leading to an overall immune-suppressive state [21,22,23]. This is another key feature of the GBM internal microenvironment. MES-phenotype GBM typically shows more abundant immune component infiltration than other subtypes, which is related to its higher secretion of chemokines such as POSTN [24]. In the hypoxic microenvironment, the cytotoxic T lymphocytes exhibit marked T cell exhaustion, with gene expression related to the immune checkpoint pathway [25]. The most abundant TAM population not only contributes to glioma immune evasion through molecules such as PD-L1 [26] but also promotes the malignant behavior of gliomas, such as invasion and angiogenesis, through various mechanisms [27,28]. Recent single-cell sequencing studies have shown that TAMs can directly induce the MES phenotype of GBM through ligand–receptor interactions [29]. The close link between hypoxia and immune infiltration microenvironment features and the MES phenotype is also evident in the spatial dimension. In a recent spatially resolved multi-omics study of GBM, the “reactive-hypoxia” spatial transcriptional program was highly consistent with the “MES-like” region in tumor tissue sections and enriched with tumor-associated myeloid cells and T cells [25,30]. Therefore, by integrating the characteristic hypoxic and immune-infiltrated tumor microenvironment, a comprehensive analysis of GBM may help to identify key genes that regulate the acquisition of the MES phenotype.

PLAUR, the plasminogen activator, urokinase receptor, encodes a single-chain glycosylated protein with cysteine residues, known as uPAR, which is anchored to the cell membrane by a glycosyl phosphatidyl inositol (GPI) linkage. PLAUR overexpression has been observed in various tumors and linked to poor survival and prognosis [31]. Typically, uPAR binds to uPA and catalyzes the conversion of plasminogen to plasmin, which triggers a proteolytic cascade that degrades the extracellular matrix, facilitating the metastasis, invasion, angiogenesis, and growth of diverse tumors [32,33]. Moreover, PLAUR exhibits its potential in modulating the immune component in different cancers. High PLAUR expression is associated with increased M2 macrophage infiltration, indicating its role in the immune microenvironment [34]. Despite these studies revealing some of the functions of PLAUR, comprehensive investigation beyond the cell-autonomous level is scarce. Therefore, it is imperative to design a study that moves beyond the cell-intrinsic factor and incorporates the tumor-microenvironmental features to explore the correlation between PLAUR and the MES phenotype.

Therefore, this study set out to comprehensively demonstrate the correlation between PLAUR and the hypoxia immunosuppression microenvironment and investigate PLAUR’s ability to regulate the MES phenotype in terms of both cell-intrinsic and cell-extrinsic factors. The approaches for study included bioinformatics analysis, integrating bulk and single-cell sequencing, functional experiments with glioma cell lines and patient-derived primary cells, and a glioma xenograft model in BALB/c nude mice. Conclusively, PLAUR is the hub gene that correlates positively with the hypoxia and immune microenvironment, and PLAUR determines the MES phenotype of GBM as an intrinsically regulatory gene. Furthermore, PLAUR demonstrates heterogenous expression in TAMs and glioma cells, mediating the MES phenotype through cell-to-cell interaction. Our study provides new insights and a better understanding of the heterogeneity and development of promising therapeutic targets for GBM.

## 2. Materials and Methods

### 2.1. Ethics Statement

All the GBM tissue samples and matching clinical information were acquired from the Department of Neurosurgery at The Second Affiliated Hospital, Zhejiang University School of Medicine. The studies involved patients’ samples, which were collected following the Declaration of Helsinki, and the protocol was approved by the Ethics Committee of The Second Affiliated Hospital, Zhejiang University School of Medicine (approval no. 2022-1076, date of approval: 5 December 2022). The animal study protocol was approved by the Ethics Committee of The Second Affiliated Hospital, Zhejiang University School of Medicine (approval no. 2023-132, date of approval: 27 September 2023). All patients involved in this study provided their written informed consent. The clinical characteristics of patients are summarized in the Supplementary Materials, Table S1.

### 2.2. Data Acquisition and Preprocessing

We obtained gene expression data from glioma specimens from two publicly available datasets, namely The Cancer Genome Atlas (TCGA-GBM, https://portal.gdc.cancer.gov/ (accessed on 1 October 2022)) and the Chinese Glioma Genome Atlas (CGGA, http://www.cgga.org.cn/ (accessed on 1 October 2022)). All sequencing data were converted to TPM format for downstream analyses. Important clinical information that was not available on the official website was obtained from the GlioVis database (http://gliovis.bioinfo.cnio.es/ (accessed on 1 October 2022)). After excluding the samples without prognostic information and with a survival time of less than 30 days, 152 GBM patients from TCGA and 305 glioma patients (LGG + GBM) from CGGA were included in the study. We also evaluated the gene signatures of samples from different GBM pathological structures using the Ivy Glioblastoma Atlas Project (IvyGAP). In addition, single-cell RNA-seq (scRNA-seq) data were downloaded from GSE131928 [13]. A total of 5742 cells from primary IDH-WT adult GBM patients’ scRNA-seq data based on the Smart-seq2 protocol were extracted from the GEO database (https://www.ncbi.nlm.nih.gov/geo (accessed on 1 November 2022)).

### 2.3. Identification of Hypoxia-Immunosuppression-Related Differentially Expressed Genes

To identify the hypoxia status, a hypoxia signature was used according to a prior study involving pan-cancer analysis [35]. By using the “ConsensusClusterPlus” R package (1.60.0), unsupervised hierarchical clustering was employed to divide GBM patients into three distinct clusters, similar to the procedure in previous research [35]. Then, we implemented the ssGSEA algorithm in the “GSVA” R package (1.46.0) to calculate the hypoxia score of each GBM patient, which helped us to assess the hypoxia status, including “hypoxia^high^ (HY^high^)”, “hypoxia^intermediate^ (HY^intermediate^)”, and “hypoxia^low^ (HY^low^)” groups. To explore the tumor immune microenvironment of each patient, we quantified the levels of 28 immune cells by ssGSEA [36]. The scores of these 28 immune cells were categorized into high and low groups based on the median. We performed univariate Cox regression analysis to screen immune cells associated with prognosis (*p* < 0.05). Then, GBM patients were grouped into “immunosuppression^high^ (IM^high^)” and “immunosuppression^low^ (IM^low^)” based on the infiltrating status of MDSCs. The above hypoxia and immunosuppression status was further combined into a two-dimensional index and GBM patients were segmented into three groups, namely “HY^high^-IM^high^”, “HY^low^-IM^low^”, and mixed groups. The “limma” R package (3.52.4) was used to obtain the differentially expressed genes (DEGs) between groups. Only genes with both a *p* value < 0.05 and an absolute value of log2 (fold change) > 2 were regarded as HY-IM-related DEGs. Moreover, we applied the functions in the “tinyarray” R package (2.2.7) to obtain heatmaps and volcano maps of the data.

### 2.4. WGCNA Construction

WGCNA (version 1.71), a systems biology approach, is used to construct weighted gene co-expression networks that can identify modules of highly collaborative genes based on the correlation between gene sets and phenotypes [37]. We selected the top 5000 genes with the greatest variation in the transcriptome data of the GBM patients and subsequently performed sample clustering, setting a threshold of 90 to remove outlier samples. By using the function “pickSoftThreshold”, the soft threshold value of the correlation matrix was selected as 9. We used the adjacency matrix to calculate the topological overlap measure (TOM), which measures the connectivity among all the genes in the network. Based on the TOM, we conducted hierarchical average linkage clustering, which grouped genes with similar patterns of expression. The dynamic tree cutting method was used to identify different modules, with a minimum module number of 30. Then, the parameter mergeCutHeight was set at 0.3 to merge similar modules. The correlation between ultimate modules and phenotypes was further analyzed. Based on the highest correlation between hypoxia and immunosuppression, we selected the brown module for further study.

### 2.5. Identification of the Hub Gene in Hypoxia-Immunosuppression-Related Gene Set

By taking the intersection between HY-IM-related DEGs and brown module genes, we obtained a HY-IM-related gene set containing 163 genes. Univariate Cox regression was conducted to identify prognosis-associated genes (*p* < 0.05). Furthermore, Lasso–Cox regression analysis was performed using the “glmnet” R package (4.1–6) and tenfold cross-validation was carried out to select the minimal penalty term (λ). Based on the coefficients of the optimal genes, we selected the PLAUR gene with the highest coefficient as the hub gene for further analyses.

### 2.6. Differential Expression and Functional Enrichment Analysis

According to the median of PLAUR expression, the patients were divided into PLAUR^high^ and PLAUR^low^ groups. PLAUR-associated DEGs were identified using the “limma” R package for *p* values < 0.05 and a log-fold change absolute value > 1. These DEGs were chosen for functional annotation, such as gene ontology (GO), via the “ClusterProfiler” R package (4.4.4). We downloaded the gene set of “h.all.v2022.1.Hs.symbols.gmt” from MSigDB to perform GSVA using the “GSVA” R package (1.44.5). In addition, we also conducted the mesenchymal and proneural subtype of GBM enrichment analysis by GSEA (https://www.gsea-msigdb.org/gsea/index.jsp (accessed on 15 October 2022)).

### 2.7. Assessment of the Immune Response and Mesenchymal Subtype

XCell was applied to assess the status of immune infiltration. The ESTIMATE algorithm was used to calculate three indices, namely the immune, stromal, and estimate scores. Tumor purity scores were inferred by the following formulae [38]. In addition, Pearson’s correlation analysis was performed to determine the link between the PLAUR expression levels and immune checkpoint genes. Tumor purity score = cos (0.6049872018 + 0.0001467884 × ESTIMATE score).

To clarify the relationship between PLAUR and the GBM mesenchymal subtype, we calculated the correlation coefficients between the PLAUR’s expression level and mesenchymal markers (CD44, CHI3L1, VIM, and ANXA1) as well as proneural markers (ASCL1, DLL3, OLIG2, and CDH1), which were visualized using the “corrplot” R package (0.92).

### 2.8. Single-Cell Data Analysis and Cell-to-Cell Interaction Analysis

We downloaded the single-cell RNA sequencing (scRNA-seq) data from the dataset GSE131928. Based on the previous literature [13], we performed scRNA-seq analysis using the R package (Seurat 4.3.0). After the quality control process and normalization, highly variable genes were obtained using Seurat’s “FindVariableFeatures” function. Then, principal components analysis (PCA) was performed for dimensionality reduction and 10 PCA dimensions were selected for t-SNE clustering. DEGs for different clusters were identified and cell types were annotated using different cell surface markers acquired from the official CellMarker website (http://biocc.hrbmu.edu.cn/CellMarker/ (accessed on 10 November 2022)). Subsequently, we performed tumor cell status annotation. GBM tumor cells were extracted from the first cell annotation results for normalization and t-SNE clustering. The meta modules published [13] were applied to further assess GBM cellular states via “AddModuleScore” of Seurat. The expression distribution of PLAUR among clusters was visualized by dot plots. As MES cells have different features, we extracted MES-like cells and calculated the expression of different signatures by “AddModuleScore”. Then, correlation analysis was performed to assess the implications of PLAUR in MES-like cells. For the cell-to-cell interactions, “NicheNet” (1.0.0) analysis [39] was applied to link ligands secreted by the tumor-associated macrophages (TAMs) to MES-like marker genes. We first downloaded the ligand–target prior model, a database for ligand–receptor networks, and a weighted integrated network (https://zenodo.org/record/3260758 (accessed on 15 November 2022)). We considered the genes that were detected in at least 10% of cells among TAMs or tumor cells as expressed genes. Next, the ligand activity was computed compared to the background set of genes, and we ranked the ligands according to the presence of ligand target genes in the gene set of interest. Finally, we visualized the optimal ligand-binding receptors and the potential to regulate target genes via heatmaps.

### 2.9. Cell Culture and Reagents

Human GBM cell lines A172, LN229, and U251 (the transcriptional subtype was classified as MES, PN, and CL, respectively) and human monocyte cell line THP-1 were purchased from the Chinese Academy of Sciences Cell Bank. The transcriptional subtype of GBM cell lines was classified by the ssGSEA method [9] using sequencing data acquired from the Cancer Cell Line Encyclopedia (CCLE) database (https://sites.broadinstitute.org/ccle (accessed on 1 February 2023)). All the GBM cell lines were cultured in DMEM (Thermo Fisher Scientific, Waltham, MA, USA) supplemented with 10%FBS (Biological Industries, Kibbutz Beit Haemek, Israel) and 1% Penicillin/Streptomycin (P/S, Thermo Fisher Scientific, Waltham, MA, USA). THP-1 was cultured with RPMI 1640 medium (Thermo Fisher Scientific, Waltham, MA, USA) with 10%FBS and 1% P/S and treated with 200 ng/mL PMA (Sigma-Aldrich, St. Louis, MO, USA) for 24 h to induce the differentiation of macrophages. The isolation and culture of the patient-derived glioblastoma cells were applied to establish the primary GBM cell line “GBM129” in our study. The detailed method was described in the work of Hara et al. [29]. Briefly, tumor tissue from a newly diagnosed GBM patient was dissociated with an enzyme and then cultured in neurobasal medium (Thermo Fisher Scientific, Waltham, MA, USA) supplemented with 1/2× N2 (Thermo Fisher Scientific, Waltham, MA, USA) and 1 x B27 (Thermo Fisher Scientific, Waltham, MA, USA), 1% P/S, 1.5× Glutamax (Thermo Fisher Scientific, Waltham, MA, USA), 20 ng/mL of human recombinant EGF (R&D Systems, Minneapolis, MN, USA), and 20 ng/mL of human recombinant bFGF (R&D Systems, Minneapolis, MN, USA). All cells were authenticated by short tandem repeat (STR) analysis before the experiments.

### 2.10. PLAUR mRNA Interfering and Overexpression

For the transient knockdown of PLAUR, two independent sequences of siRNA and a corresponding negative control purchased from the GenePharma company (Shanghai, China) were used according to the recommended protocol of lipofectamine 3000 (Thermo Fisher Scientific, Waltham, MA, USA), and cells were harvested for the subsequent experiments 48 h after transfection. The persistent knockdown of PLAUR and the negative control was achieved by a lentivirus expressing shRNA (LV-shPLAUR and LV-shNT) constructed by the GenChem company (Shanghai, China). Detailed information about the target sequence is provided in the Supplementary Materials, Table S2. For the overexpression of PLAUR, the sequence from NCBI (Gene ID: 5329) was inserted into the pEX-3 vector to construct the plasmid. Lipofectamine 3000 was used to transfect PLAUR or vector control plasmids into cells following the manufacturer’s protocol.

### 2.11. Immunohistochemistry (IHC), Immunofluorescence (IF), and Western Blotting (WB)

In our study, tissues resected from GBM patients were fixed with 4% paraformaldehyde (PFA) and then subjected to paraffin and frozen sections for IHC and IF staining, respectively. Protein extracts from GBM cells lines were used for WB. The detailed protocols were described previously [40]. The Fiji software was applied for the statistical analysis of the IF results (https://fiji.sc/ (accessed on 1 December 2022)). Information on the antibodies and reagents used is detailed in the Supplementary Materials, Table S3. The uncropped images of WB are also included in the Supplementary Materials, Original Images for Blots.

### 2.12. RNA Extraction and qRT-PCR

Total cell RNA was extracted using TRIzol reagent (Thermo Fisher Scientific, Waltham, MA, USA) according to the manufacturer’s protocol. Reverse transcription and qRT-PCR were performed as previously reported [41]. Information about primers is available in the Supplementary Materials, Table S2.

### 2.13. Transwell and Wound Healing Assay

After the knockdown or overexpression of PLAUR, the motility of A172 and LN229 cells was detected by a transwell assay and wound healing assay. The procedure was performed according to a previously described protocol [42]. Migrated cells and areas were quantified with the Fiji software (accessed on 1 December 2022).

### 2.14. Tumor Spheroid Invasion Assay for Patient-Derived Glioblastoma Cells

The tumor spheroid invasion assay was conducted with the 96-Well 3D Spheroid BME Cell Invasion Assay reagent kit (Trevigen, Gaithersburg, MD, USA) according to the manufacturer’s instructions. The detailed protocol was described previously [43]. Briefly, 2 × 10^4^ patient-derived glioblastoma cells GBM129 were incubated with the spheroid formation matrix for 48 h to generate tumor spheroids. Then, the spheroids were embedded into the invasion matrix with conditioned medium (CM) from different THP-1 macrophages, setting fresh medium as a control. Tumor spheroids were photographed at time points of 0, 3, and 6 days after CM treatment by a fluorescence microscope (Leica, Wetzlar, German). Referring to the CM acquisition, THP-1 cells were transfected with LV-shPLAUR or LV-shNT. After differentiation into macrophages induced by PMA, fresh culture medium for patient-derived glioblastoma cells (neurobasal medium with supplements) was added at 24 h to obtain the THP-1 macrophage CM. The floating cells and debris of CM were removed by centrifugation at 3000× *g* for 20 min, and then the CM was filtered with a 0.22 μm filter (Millipore, Burlington, MA, USA).

### 2.15. Flow Cytometry of THP1-Derived Macrophages

THP1 monocytes were seeded into 6-well plates and induced to macrophages with PMA, and then the culture plates were rinsed with PBS. Macrophages in wells were transfected with 2 ug PLAUR plasmid (vector as control) following the method described before. Then, 48 h after transfection, macrophages were harvested by EDTA–trypsin (Thermo Fisher Scientific, Waltham, MA, USA) and washed with PBS. Then, the conjugated antibodies were added to stain the cells on ice for 30 min. Next, the expression of CD11b and CD163 on macrophages was detected by a CytoFLEX flow cytometer (Beckman Coulter, Brea, CA, USA). The information on the antibodies and reagents used is detailed in the Supplementary Materials, Table S3.

### 2.16. Animal Study

To investigate the effect of PLAUR on tumor growth in vivo, the intracranial xenograft model was applied. Here, 4-week-old male BALB/c nude mice (Gempharmatech, Nanjing, China) were bred under specific-pathogen-free conditions at 24 °C on a 12-h day–night cycle for the establishment of the animal model. We labeled the constructed LV-shPLAUR and LV-shNT GBM abovementioned with luciferase using a lentivirus. Then, 5 × 10^5^ luciferase-labeled GBM cells were implanted into the right frontal lobe of each mouse. Tumor volume was measured by using the IVIS Lumina Series III (PerkinElmer, Waltham, MA, USA) after 150 mg/kg D-luciferin (PerkinElmer, Waltham, MA, USA) intraperitoneal injection. All procedures that involved animals were conducted according to ethical policies and procedures approved by the Ethics Committee of The Second Affiliated Hospital of Zhejiang University School of Medicine.

### 2.17. Statistical Analysis

Statistical analysis was conducted using SPSS 20.0 and GraphPad Prism 9. All data are expressed as means ± SDs unless otherwise specified. For normally distributed data, Student’s *t*-tests and one-way ANOVA were utilized for comparisons between two independent groups and among multiple groups, respectively. In cases of non-normally distributed data, the Mann–Whitney U test and the Kruskal–Wallis test were employed for comparisons. Survival curves were generated using the Kaplan–Meier method, and differences between survival curves were assessed using the log-rank test. A significance level of *p* < 0.05 was considered indicative of statistical significance. *p* values are indicated as follows: * *p* < 0.05; ** *p* < 0.01; and *** *p* < 0.001.

## 3. Results

### 3.1. Identification of HY-IM-Related DEGs in GBM

**Figure 1:**
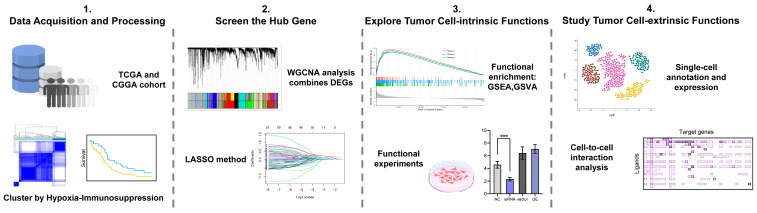
The experimental design and workflow of the current study. (1) The glioma sequencing data were acquired from the TCGA and CGGA databases and were processed for clustering by the hypoxia and immunosuppression features. (2) The hub gene was screened by an integrative strategy combing WGCNA analysis, hypoxia-immunosuppression-related DEGs, and the Lasso algorithm. (3) The tumor cell-intrinsic functions of the hub gene were explored through bioinformatical analysis and functional experiments. (4) The tumor cell-extrinsic functions of the hub gene were studied by analyzing the single-cell data and cell-to-cell interactions. *** *p* < 0.001.

**Figure 2:**
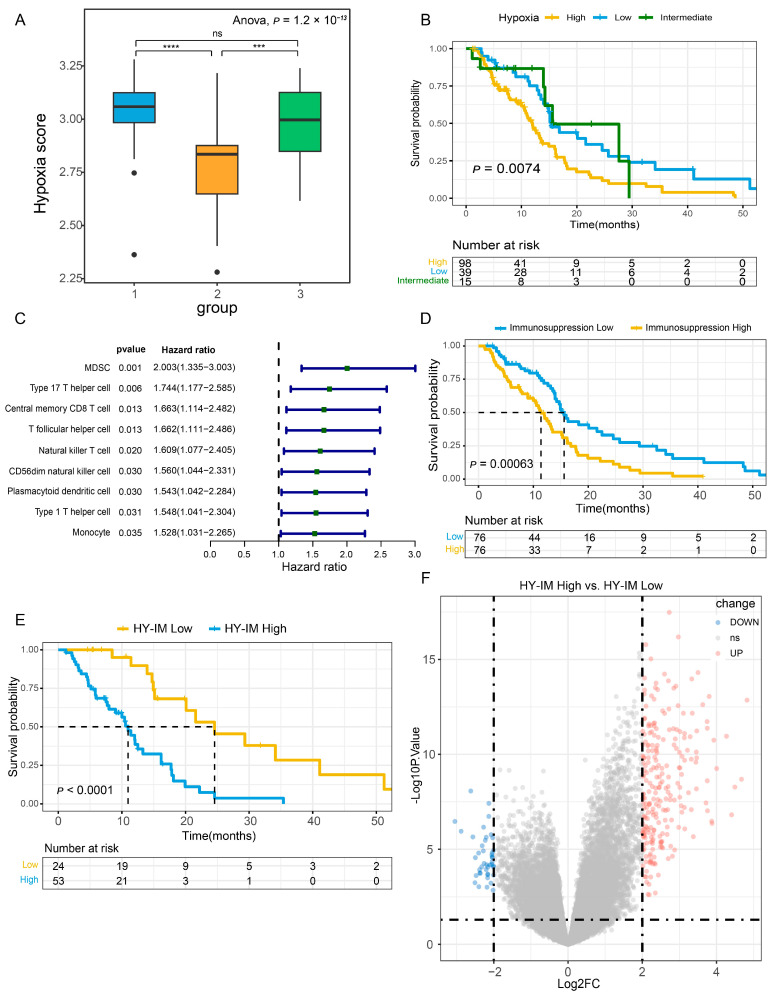
Identification of hypoxia immunosuppression status in GBMs. (**A**) TCGA-GBM patients were grouped based on hypoxia signature score. (**B**) Survival analysis for different hypoxia conditions. (**C**) Univariate Cox regression analysis for 9 prognosis-related immune cells. (**D**) Survival analysis for IM^high^ and IM^low^ groups. (**E**) Survival analysis for HY^high^-IM^high^ and HY^low^-IM^low^ groups. (**F**) Volcano plot of DEG analysis between HY^high^-IM^high^ and HY^low^-IM^low^ groups. ns, not significant, *** *p* < 0.001, **** *p* < 0.0001.

The flowchart of our study design is shown in Figure 1. We classified 152 GBM patients from the TCGA database into three hypoxia groups based on a 15-gene hypoxia gene set that was considered as the best performer for the assessment of a tumor hypoxia condition (Figure S1A). According to the hypoxia score, we redefined the three groups as HY^high^ (*n* = 98), HY^intermediate^ (*n* = 15), and HY^low^ (*n* = 39) (Figure 2A). We observed that hypoxia was associated with a worse prognosis (Figure 2B, log-rank test, *p* = 0.0074). Hypoxia-related DEGs were obtained using the limma R package, and GO enrichment analysis showed significant enrichment in terms related to leukocyte activities (Figure S1B,C).

To evaluate the immune infiltration of TCGA-GBM samples, the ssGSEA algorithm was applied for the deconvolution analysis of 28 immune cell types in GBM bulk sequencing (Table S4). The cutoff point for high and low infiltration was determined by the median scores. Then, we conducted univariate Cox regression on 28 immune cells, and nine prognosis-related infiltrating immune cells were found (HR > 1, *p* < 0.05) (Figure 2C). Of these, MDSC had a potent immunosuppressive ability. Therefore, the IM^high^ and IM^low^ groups were identified according to the extent of MDSC infiltration status. A survival analysis demonstrated that patients with high MDSC infiltration exhibited the worst prognosis (log-rank test, *p* = 0.00063) (Figure 2D). Immunosuppression-related DEGs were obtained by the limma R package, and GSEA analysis showed the positive enrichment of the HIF-1 signaling pathway in the IM^high^ group, indicating an association between immunosuppression and the hypoxia status in GBM (Figure S1D,E).

Based on the identified hypoxia and immunosuppression statuses, we categorized patients into three groups: “HY^high^-IM^high^”, “HY^low^-IM^low^”, and a mixed group. The survival analysis demonstrated that patients in the HY^high^-IM^high^ group exhibited a significantly worse prognosis compared to those in the HY^low^-IM^low^ group (log-rank test, *p* < 0.0001, Figure 2E). Furthermore, a total of 285 HY-IM-related DEGs were identified by the limma R package between the HY^high^-IM^high^ and HY^low^-IM^low^ groups (Figure 2F, Figure S1F). GSEA analysis showed the functional enrichment of the terms “negative regulation of immune system process” and “response to hypoxia” (Figure S1G). Taken together, our data suggest that a robust association between hypoxia and immunosuppression features exists in the GBM microenvironment, leading to an unfavorable prognosis for patients.

### 3.2. Detection of the HY-IM-Related Key Module by WGCNA

**Figure 3:**
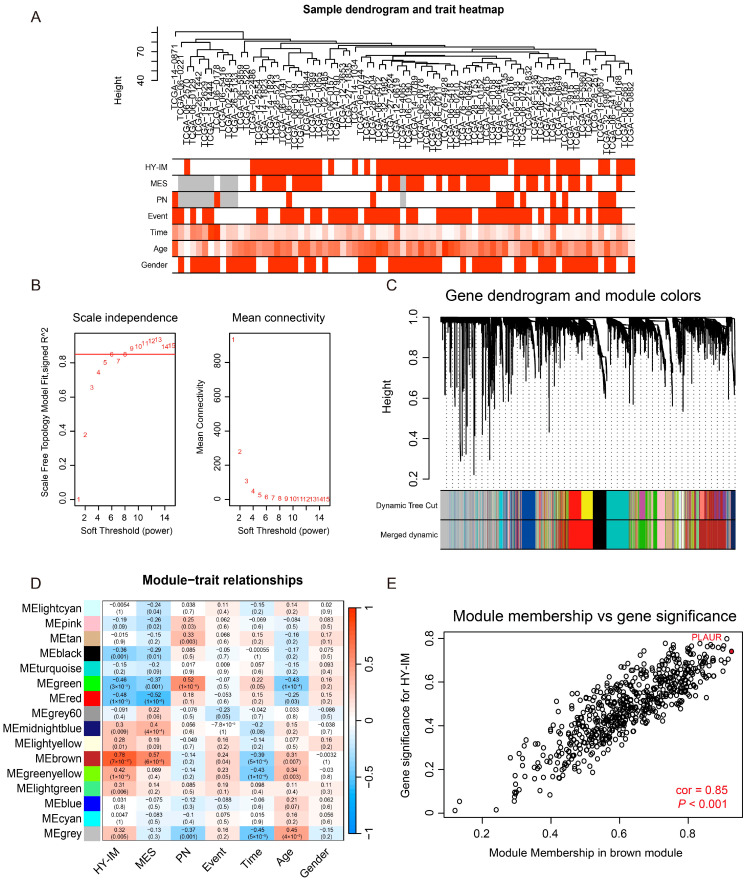
Detection of the key module for HY-IM features by WGCNA. (**A**) Dendrogram and trait heatmap for included samples. (**B**) Setting of the soft threshold power to achieve a scale-free network. (**C**) Co-expressing modules were clustered based on the expression patterns of genes. (**D**) Heatmap of correlations between modules and traits. (**E**) Scatter plot of correlations between gene significance and module membership in brown module.

To find the key modules associated with hypoxia and immunosuppression features, we performed WGCNA analysis on HY^high^-IM^high^ patients and HY^low^-IM^low^ patients. We selected “90” as the criterion to remove atypical samples (Figure 3A). The soft-thresholding power was set as “9” (scale-free R^2^ = 0.85) and a scale-free network was achieved (Figure 3B). After merging similar modules, 16 gene co-expression modules were identified using the dynamic tree cut method (non-clustering genes shown in gray) (Figure 3C). A heatmap of module and sample trait correlations was created, and it was determined that the brown module was highly correlated with hypoxia and immunosuppression (Figure 3D). Surprisingly, the brown module was also highly correlated with the GBM MES subtype (Figure 3D); this was consistent with previous studies and suggested that the MES subtype shared hypoxic and immunosuppressive properties [9]. The scatter plots concerning gene significance vs. module membership in the brown module showed identical results (Figure 3E). Eventually, the brown module (612 genes) was considered as the HY-IM-related key module.

### 3.3. Identification of PLAUR as the Hub Gene of HY-IM Feature in GBM

**Figure 4:**
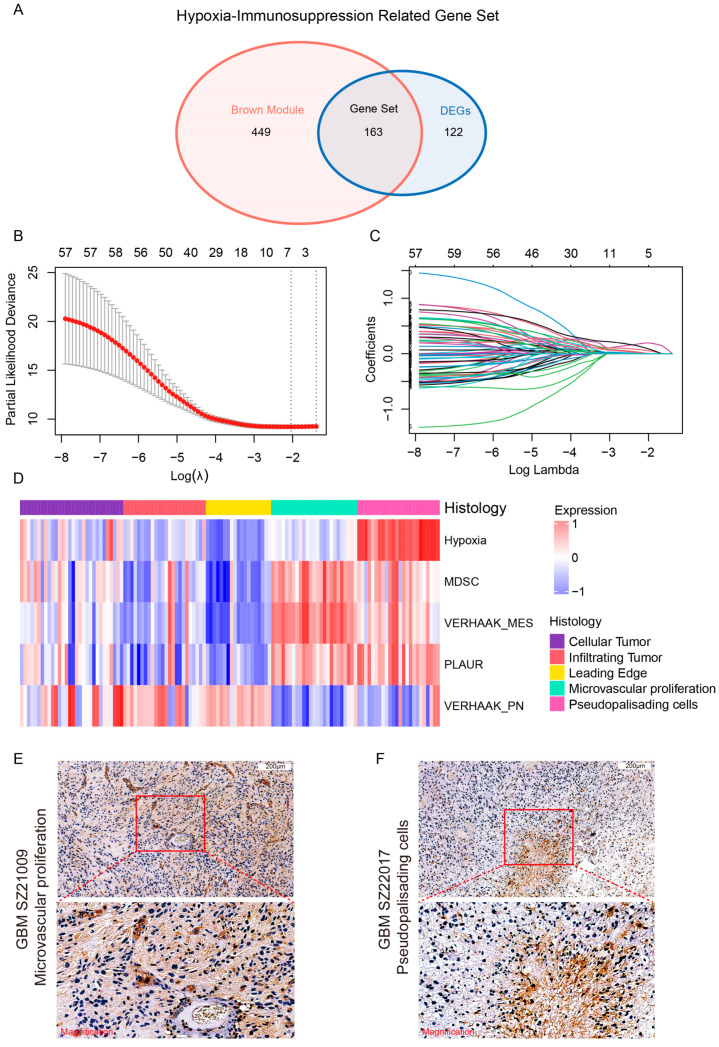
Identification of PLAUR as the hub gene. (**A**) Construction of hypoxia-immunosuppression-related gene set. (**B**,**C**) Lasso–Cox regression model to select the hub gene of the gene set. (**D**) Heatmap of the PLAUR expression or gene set score sorted by the histologic features defined by ivyGAP. (**E**) Representative IHC image with region magnifying PLAUR expression in the MVP structure. (**F**) Representative IHC image with region magnifying PLAUR expression in the PAN structure. Scale bar, 200 μm.

By intersecting HY-IM-related DEGs and key module genes, we obtained a HY-IM-related gene set containing 163 genes (Figure 4A). To further identify genes associated with prognosis, we used univariate Cox analysis for screening and retained 59 genes with *p* < 0.05 (Table S5). Next, the Lasso–Cox regression method was applied to obtain five optimal genes (PLAUR, HSPA7, MT1H, PODNL1, and PDZK1IP1) with minimum deviation in the score and a best λ of 0.1302 (Figure 4B,C). PLAUR was considered as the hub gene for further analysis because it achieved the highest coefficient (0.19580) in the Lasso–Cox regression model (Table S6). Interestingly, we also found that PLAUR had high module membership (MM = 0.93) and high gene significance (GS = 0.74) in the brown module (Figure 3E).

Next, we sought to explore the correlation between PLAUR and the clinicopathological characteristics of GBMs. Firstly, GBM patients from the TCGA cohort were categorized into PLAUR^high^ and PLAUR^low^ groups based on the median expression of PLAUR. Among these, the GBM subtype displayed differential distribution patterns (Figure S2A), and the PLAUR expression level was highest in the MES subtype of GBM (Figure S2B). Furthermore, we investigated the association between PLAUR expression and clinical phenotypes in the CGGA-325 datasets, including LGG and GBM. Evidently, we observed that PLAUR expression was significantly upregulated in the higher grades (Figure S2C,D). The IHC staining for the PLAUR protein using tissue sections from glioma patients validated the bioinformatic result (Figure S2E,F). The survival analysis for patients in the two databases demonstrated that the high expression of PLAUR produces the worst prognosis (Figure S2G,H).

Subsequently, the ssGSEA algorithm was used to analyze the expression of PLAUR in distinct histological structures defined by the Ivy Glioblastoma Atlas Project (ivyGAP). We found that PLAUR was highly expressed in “microvascular proliferation” (MVP) and “pseudopalisading cells around necrosis” (PAN) structures (Figure 4D). The heatmap further revealed a consistent expression pattern of PLAUR with hypoxia, immunosuppression, and the MES subtype in relation to histological features; this pattern was not seen in the PN subtype (Figure 4D). IHC staining demonstrated the abundant expression of PLAUR in MVP and PAN structures within GBM tissues (Figure 4E,F). The above data confirmed that PLAUR, as the hub gene, was associated with a hypoxic and immune microenvironment in GBM. Therefore, we speculated that PLAUR could play crucial roles in the immune and mesenchymal phenotype.

### 3.4. PLAUR Is Associated with Immune Microenvironment of GBM

**Figure 5:**
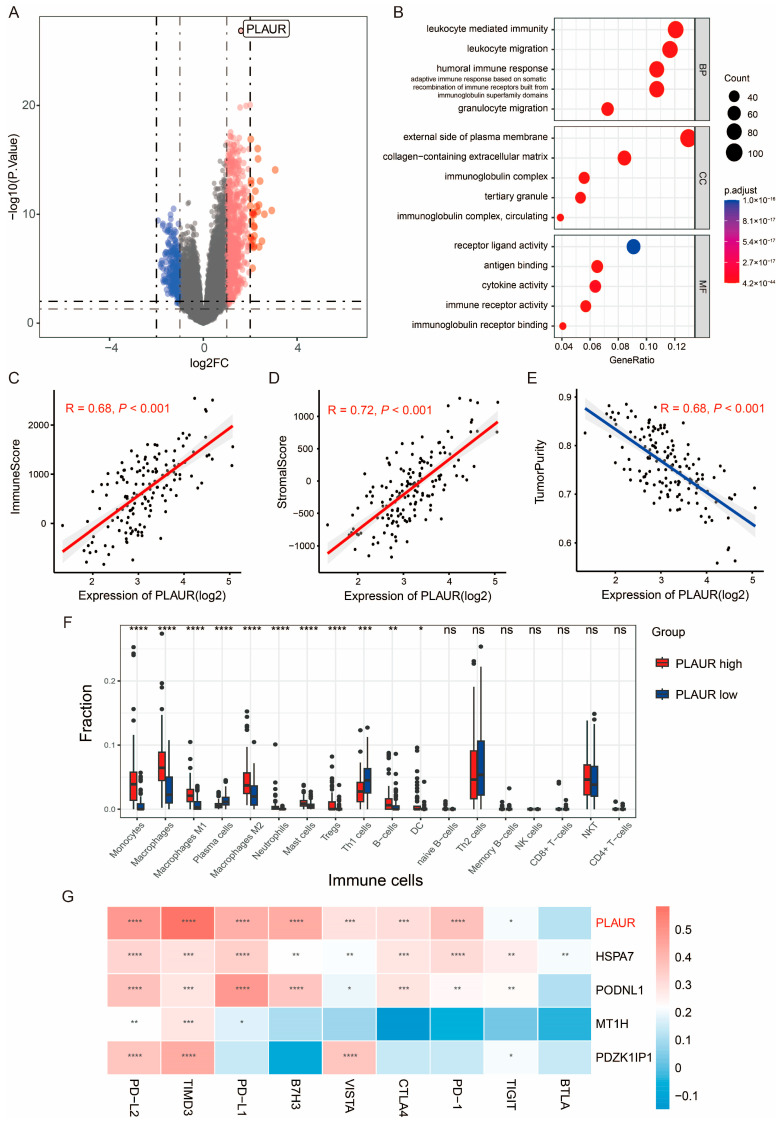
PLAUR is associated with the immune microenvironment of GBM. (**A**) Volcano plot of DEGs between PLAUR high and low groups. (**B**) GO enrichment analysis shows involvement of PLAUR in immune functions. (**C**–**E**) Correlation analysis between PLAUR expression and immune score, stromal score, and tumor purity in TCGA-GBM, respectively. (**F**) Plot of differential immune fraction in PLAUR high and low groups. (**G**) Correlation heatmap of expression between selected gene and immune checkpoint members. ns, not significant, * *p* <0.05, ** *p* < 0.01, *** *p* < 0.001, **** *p* < 0.0001.

By using the criteria |log2 FC| > 1 and *p* value < 0.05, we obtained 1031 DEGs (744 upregulated and 287 downregulated) between the PLAUR^high^ and PLAUR^low^ groups in TCGA-GBM (Figure 5A). The GO analysis indicated that immune-response-related biological processes were enriched (Figure 5B). Hence, we calculated the immune, stromal, and tumor purity scores by adopting the ESTIMATE method. The PLAUR expression level showed a positive correlation with the immune score (R = 0.68, *p* < 0.001) and stomal score (R = 0.72, *p* < 0.001) and a negative correlation with tumor purity (R = 0.68, *p* < 0.001) (Figure 5C–E). In addition, we used the xCell algorithm to estimate the immune fraction in GBM bulk sequencing. We found that the PLAUR^high^ group tended to show a higher level of M2 macrophages and Treg cells (Figure 5F). The immunofluorescence staining and the corresponding statistics for PLAUR, CD163, and CD206, using frozen sections of GBM tissues, demonstrated close spatial proximity between the PLAUR protein and M2 macrophages (Figure S3A–D). To investigate the effect of PLAUR on M2 macrophage polarization, flow cytometry was applied to detect the CD11b+ and CD163+ cells in macrophages transfected with PLAUR or vector plasmids. As the plots indicated, PLAUR overexpression increased the CD163-positive rate of macrophages without any other exogenous stimulates (Figure S3E).

Considering the crucial role of immune checkpoint molecules in immune evasion, we further assessed the correlation between PLAUR expression and several common immune checkpoint members, such as PD-1, PD-L1, PD-L2, CTLA4, TIMD3, and B7H3. Pearson correlation analysis demonstrated that PLAUR expression was highly positively correlated with the checkpoint members (Figure 5G). Collectively, these results indicate that PLAUR is linked to the formation of the immune microenvironment in GBM.

### 3.5. PLAUR Determines the MES Phenotype of GBM and Tumor Progression In Vivo

**Figure 6:**
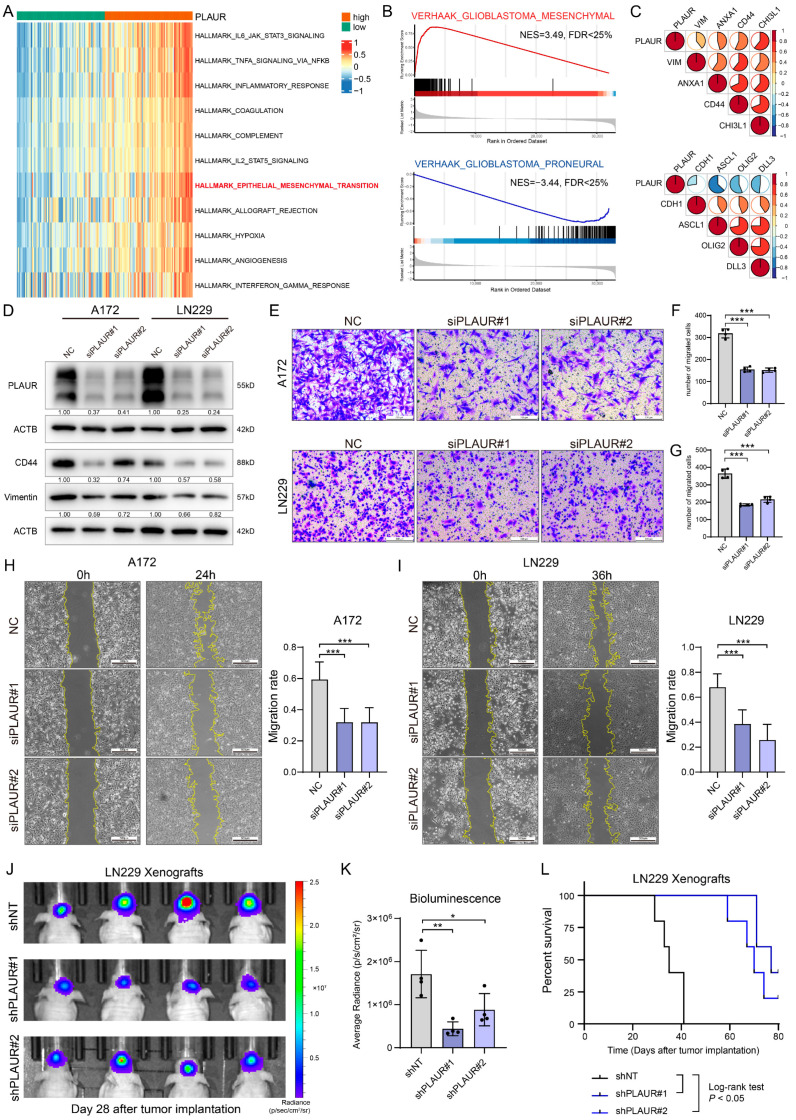
PLAUR regulates the mesenchymal phenotype and tumor growth of glioma. (**A**) GSVA analysis in TCGA-GBM indicates that PLAUR is associated with several biological processes, including EMT. (**B**) GSEA analysis shows that PLAUR expression is positively associated with MES subtype and negatively with PN subtype. (**C**) Pie plot of correlation between PLAUR expression and selected MES and PN marker genes. (**D**) Western blot analysis for CD44 and vimentin expression with ACTB as endogenous control after PLAUR silencing with siRNA in A172 and LN229. (**E**) Representative images of transwell assay for A172 and LN229 cells with PLAUR silencing. Scale bar = 200 μm. (**F**,**G**) Statistical analysis of transwell assay for A172 and LN229, respectively. (**H**,**I**) Representative images of wound healing assay with statistical analysis for A172 and LN229 cells after PLAUR silencing, respectively. Scale bar = 500 μm. (**J**) Bioluminescence imaging for xenografts constructed by shNT-LN229 and shPLAUR-LN229 at 28 days after tumor implantation. (**K**) Statistical analysis for the bioluminescence imaging in (**J**). (**L**) Survival curve for mice in sh-NT and sh-PLAUR groups; statistical significance was tested by log-rank test. * *p* <0.05, ** *p* < 0.01, *** *p* < 0.001.

Subsequently, we performed a GSVA analysis using TCGA-GBM data to explore other biological processes regulated by PLAUR. As depicted in the heatmap, in addition to the hypoxia- and immune-related signal pathways, a positive association with epithelial–mesenchymal transition (EMT) was observed (Figure 6A). The GSEA analysis of the VERHAAK_MESENCHYMAL and VERHAAK_PRONEURAL gene sets yielded consistent results (Figure 6B), suggesting that PLAUR may regulate the mesenchymal phenotype of GBM. Following this, a correlation analysis was conducted between PLAUR and marker genes’ expression in the GBM dataset. In line with the functional enrichment, PLAUR expression exhibited a positive correlation with MES markers and a negative correlation with PN markers (Figure 6C). To validate the bioinformatics findings, we employed small interfering RNA (siRNA) to investigate the effects of PLAUR on glioma cell lines. Following the knockdown of PLAUR in glioma cells, the decreased expression of CD44 and vimentin was detected by Western blotting, while the overexpression of PLAUR via a plasmid was shown to elevate the protein level (Figure 6D, Figure S4A,B).

Tumor cell motility was closely associated with the mesenchymal phenotype. Herein, we employed transwell and wound healing assays to investigate the impact of PLAUR on cell motility. These experiments confirmed that siRNA-mediated interference with PLAUR expression inhibits tumor migration significantly (Figure 6E–I). Conversely, PLAUR overexpression promoted tumor migration (Figure S4C–J). To assess the impact of PLAUR on tumor growth in vivo, we established an intracranial xenograft model using lentiviral shPLAUR-transfected LN229 and U251, with shNT as a negative control. The knockdown of PLAUR expression with lentiviral shRNA was confirmed by Western blotting (Figure S4K). Bioluminescence measured at specific timepoints indicated that PLAUR knockdown attenuated tumor growth in mice (Figure 6J,K, Figure S4L). The survival analysis further proved that silencing PLAUR expression prolonged the survival of the experimental animals (Figure 6L, Figure S4M). Taken together, these results suggest that PLAUR could determine the MES phenotype of GBM as a tumor cell-intrinsic gene and support tumor progression in vivo.

### 3.6. Single-Cell Analysis Reveals Heterogenous Expression of PLAUR

**Figure 7:**
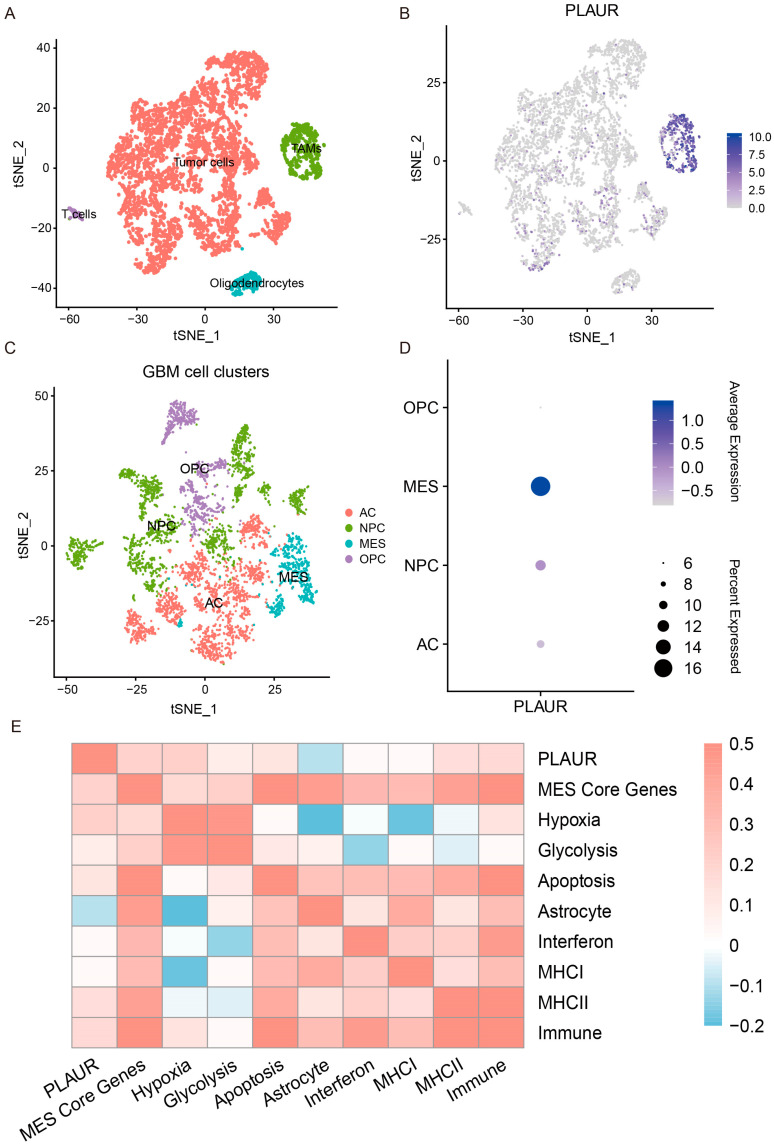
Single-cell analysis for PLAUR expression in GBM. (**A**) Annotation of four major cell types in single-cell data. (**B**) t-SNE plot of PLAUR expression in different cells in GBM. (**C**) Annotation of four cellular states in GBM cell cluster. (**D**) Dot plot of expression of PLAUR in each cellular state of GBM cells. (**E**) Heatmap depicts the Pearson correlation between PLAUR and function-specific programs in the MES-like cellular state.

To understand the heterogenous expression of PLAUR at the single-cell level, scRNA-seq data from primary IDH-WT adult GBM patients were selected for analysis (GSE131928). The Seurat R package (4.3.0) was used for scRNA-seq analysis. After quality control, standardization, dimensionality reduction, and t-SNE clustering of the samples, each type of cell cluster in GBM was annotated according to the molecular markers of particular cell types from the official CellMarker website. The t-SNE plots showed that cells in 13 clusters were categorized into four main categories: tumor cells, TAMs, oligodendrocytes, and T cells (Figure S5A, Figure 7A). The corresponding markers used for cell annotation are displayed as a dot plot (Figure S5B). We found that most TAMs and some tumor cells expressed PLAUR (Figure 7B). To investigate the PLAUR expression in distinct tumor cellular states, 4908 malignant cells were extracted for the subsequent subclustering of cellular states. We obtained 19 prominent cell subclusters, which were categorized into four main clusters (NPC-like; OPC-like; AC-like; MES-like) according to their highest scores for GBM cellular states (Figure S5C,D, Figure 7C). We found that the PLAUR mRNA exhibited the highest expression in the MES-like cellular state, aligning with previous findings of sequencing for bulk tumors (Figure 7D, Figure S5E). In addition, multiple studies have proposed that an MES-like state has distinct functional annotations, which were defined by function-specific programs [44]. Next, we explored the correlations between the expression of PLAUR and function-specific programs in the MES-like cellular state. We found that the expression level of PLAUR was positively correlated with most function-specific programs, including MES core genes, hypoxia, glycolysis, apoptosis, MHC I, MHC II, and immune, suggesting that PLAUR was associated with the hypoxia status and immune properties defined by the subclasses of the MES-like cellular state (Figure 7E). In summary, the single-cell analysis revealed that PLAUR was expressed predominantly in both MES-like glioma cells and macrophages, contributing to the heterogeneity of GBM.

### 3.7. Cell-to-Cell Interaction Analysis Reveals the Enhanced MES Driving Ability of PLAUR^high^ TAMs

**Figure 8:**
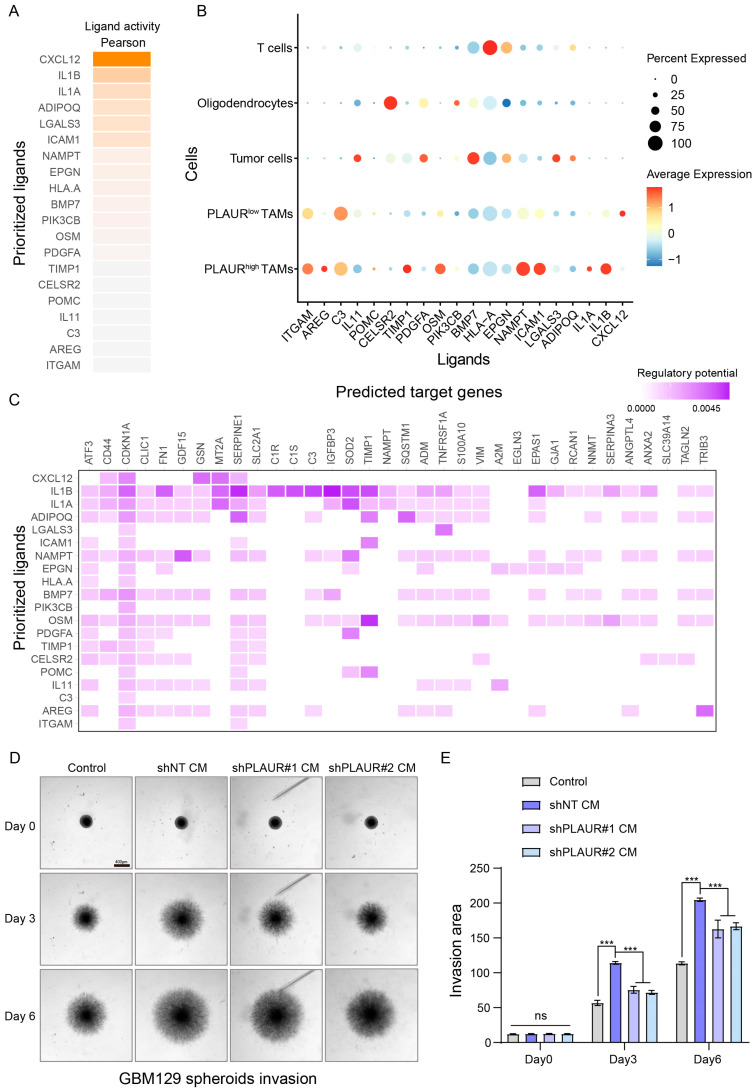
PLAUR regulates the cell-to-cell interaction between TAMs and GBM cells. (**A**) The top 20 MES-driven ligands were screened by NicheNet analysis. (**B**) The dot plot illustrates the expression of selected ligands across cell clusters. (**C**) Matrix heatmap shows the regulatory potential of the listed ligands for MES genes. (**D**) Representative images of tumor spheroid invasion assay at specific timepoints. Scale bar = 400 μm. (**E**) Statistical analysis for the quantified invasion area among distinct groups. ns: not significant, *** *p* < 0.001.

To investigate whether PLAUR regulates the MES phenotype through tumor cell-extrinsic factors, NicheNet (1.0.0) analysis was used to link the ligands secreted by TAMs with MES marker genes. Firstly, we classified TAMs into PLAUR^high^ and PLAUR^low^ groups based on the median expression value. Then, a gene set, including 100 MES marker genes, was established for the analysis. According to the predicted results, the 20 ligands with the highest activity were selected as the best ligands for further study (Figure 8A), and the potential for interaction between ligands secreted by TAMs and receptors expressed on tumor cells was demonstrated using ligand–receptor analysis (Figure S6A). Subsequently, we found that PLAUR^high^ TAMs secreted several top ligands at a comparatively high level (Figure 8B), and these ligands could target a variety of MES marker genes, thereby inducing the MES phenotype of GBM (Figure 8C).

Among these ligands, OSM and ICAM1 have been previously identified as potent molecules inducing MES phenotype transition in glioma cells [29,45]. To investigate the association between PLAUR in macrophages and the mediation of MES transition in glioma cells with OSM or ICAM1, we assessed the mRNA expression of OSM and ICAM1 in THP1-derived macrophages following lentiviral shPLAUR or shNT transfection. As illustrated in the Supplementary Materials, the knockdown of PLAUR resulted in a notable decrease in the mRNA expression of OSM and ICAM1 (Figure S6B,C). Next, we employed a tumor spheroid invasion assay using patient-derived glioblastoma cells to assess the invasiveness of tumor cells exposed to the conditional medium (CM) from shPLAUR/shNT macrophages. Consistent with previous studies, microscopic images and statistical analysis revealed that the CM from macrophages enhanced the invasion of tumor spheroids, while silencing PLAUR partially inhibited this ability (Figure 8D,E). Cumulatively, TAMs exhibiting heightened PLAUR expression demonstrated enhanced potential to drive the MES phenotype of glioma; various ligand–receptor pairs may be involved in this expression.

## 4. Discussion

Glioblastoma (GBM) is the most common primary malignant tumor in the brain, with high invasiveness and treatment resistance. GBM patients have a median overall survival (OS) of only 14.6 months and a five-year survival rate of less than 10% after the standard therapeutic strategy, which consists of maximal safe resection surgery followed by postoperative radiotherapy and TMZ chemotherapy [46]. Other treatment strategies that attempt to prolong the survival of patients include bevacizumab therapy (targeting angiogenesis), immune checkpoint therapy, and tumor-treating fields (TTF). Although there are a large number of clinical trials attempting to address questions concerning the efficacy and safety of these treatments, it has to be admitted that these treatments still cannot resolve the high treatment resistance and high recurrence risk of GBM at their root [47].

Tumor heterogeneity is a major cause of treatment resistance. GBM exhibits heterogeneity in multiple aspects, such as the complexity and plasticity of the molecular subtype, the distinctive physical structures, and the diverse immune cell components in the microenvironment [8,13]. Furthermore, these heterogenous components are extensively interrelated and interactive. Previous studies have demonstrated that GBM with the mesenchymal phenotype demonstrates highly malignant biological behavior that is strongly associated with the hypoxic and immunosuppressive microenvironment [48]. This association is evident not only in functional experiments or sequencing data but also in the spatial distribution of pathological structures in glioma tissues [30]. In this study, we integrated the two microenvironmental features of hypoxia and immunosuppression (HY-IM module) and analyzed the bulk sequencing data of the TCGA-GBM cohort, confirming that they have a consistent distribution in the ivyGAP characteristic structure (Figure 4). Of note, our analysis of the correlation between the immune cell components in glioma tissues and prognosis revealed that the myeloid-derived suppressor cell (MDSC) component is a determinant of poor outcomes. MDSCs are a heterogenous population of cells that are co-opted by tumors and exert immunosuppressive functions in the tumor microenvironment (Figure 2). They are categorized into different subgroups based on their morphological and phenotypic characteristics, which may be considered as a part of the tumor heterogeneity [49].

Considering that the complex microenvironment within the tumor plays an essential role in constructing the heterogeneity of gliomas, we adopted an integrative strategy to screen the candidate genes for further study. One of the novelties in our approach is that we predetermined the microenvironment-related genes and then applied WGCNA combined with DEGs to identify PLAUR as the hub gene (Figure 3). Li et al. investigated the prognostic significance and co-expression network of PLAUR using the TCGA dataset, which supports the high potential for application of our working strategy [50]. PLAUR, a widely studied oncogenic molecule, promotes tumor progression by regulating biological processes, such as cell proliferation and adhesion, in various tumors, including gliomas [31]. EMT, which is closely related to the invasiveness of tumor cells, can also be regulated by PLAUR. For instance, TGF-β, a key mediator of the EMT process, can directly regulate the expression of the uPA/uPAR system, and PLAUR can subsequently respond to the TGF-β-dependent biological process [51]. Moreover, PLAUR can mediate the cleavage of the active form of plasmin, thus regulating the activation of downstream matrix metalloproteinases (MMPs), which are essential for the degradation of the extracellular matrix (ECM) [32]. Despite the limited applicability of the EMT concept to glioma, the key regulatory factors and signature genes involved resemble those of the mesenchymal phenotype shift process in glioma [52]. Hence, we deduced that PLAUR could regulate the MES phenotype in glioma tumor cells. Congruent with the data presented by Gilder et al., we used bioinformatics methods to explore the association of PLAUR with the TCGA-MES subtype [53] and validated these findings through functional experiments, which mainly demonstrated the correlation between the gene expression level and tumor MES characteristics. In addition to the role of PLAUR expression in the MES characteristics of the tumor itself, our study focused on its association with hypoxia and immune microenvironmental characteristics, as hypoxia and immune infiltration (especially macrophages) are closely associated with the acquisition of the MES phenotype in gliomas, according to previous studies [15,20]. In our further analysis, PLAUR exhibited high expression in the hypoxic physical structure (PAN structure) and was closely related to the formation of the immune microenvironment (through the enrichment of immune functions and increasing infiltration of macrophages) (Figure 4 and Figure 5).

According to the ivyGAP database, which defines the pathological structures of glioma, the PAN structure has the closest relation to the MES phenotype of glioma. Hypoxia is a prominent microenvironmental feature of the necrotic and perinecrotic regions [54]. Numerous studies have reported that hypoxia enhances the MES characteristics of tumors, and a recent study demonstrated that hypoxia induces macrophage infiltration and retention [25]. Therefore, the PAN structure can be understood as the core region associated with the tumor MES phenotype and protumor immune components [55]. For example, Rashidi et al. conceptualized the hypoxic niche surrounded by myeloid/macrophages as the central domain of creatine metabolism and as primarily linked to the MES molecular subtype. Within this specialized compartment, the transportation of creatine from myeloid to tumor cells was identified as a key factor promoting survival under hypoxia stress [56]. We adopted the concept of physical structures, such as PAN, to provide a more comprehensive description of the distribution and functional characteristics of PLAUR in GBM. However, this study of physical structures relied on public databases, and the analysis method mainly involved the correlation between gene expression levels. For future studies, we aim to apply spatial omics techniques to investigate and visualize the distribution and expression characteristics of specific molecules or immune cells in the PAN structure in situ.

In recent years, single-cell sequencing has advanced our knowledge of tumor heterogeneity. By examining the molecular subtypes of glioma at the single-cell level, we can infer that the MES phenotype of the tumor derives from a subset of cells that display the MES state, and that cells in other molecular states within the tumor may switch to the MES state under the influence of specific tumor microenvironment factors [12,57]. As we have mentioned before, single-cell data enable us to investigate the role of PLAUR in the MES phenotype in both tumor cells and immune cells. To elucidate the heterogeneity of PLAUR in tumor cells, we first defined the cell types of the published single-cell sequencing data and then extracted the tumor cell population and classified it into four single-cell states (AC-like, OPC-like, NPC-like, MES-like), based on the study of Neftel et al., and discovered that PLAUR was markedly overexpressed in the MES-like subgroup, which agreed with the bulk sequencing results. Given the predominant expression of PLAUR in the TAM population, it is imperative to analyze its role in immune cells with single-cell sequencing data. An interesting finding is that although PLAUR’s expression level was positively associated with the “M2 macrophage” component, we also observed that the PLAUR protein and the canonical M2 marker (CD163 and CD206) were adjacent or colocalized in the frozen sections of glioma tissue, but a higher M1 macrophage fraction was also detected (Figure 5, Figure S3). This may reflect the true state of the tumor TAM population, because TAMs rarely show a purely classical M2 feature, but, rather, a considerable proportion of TAMs express both canonical M1 and M2 markers [58]. In addition, we also found that the expression of PLAUR was positively correlated with MHC I, MHC II, and immune functional programs in the MES-like tumor cells (Figure 7). Therefore, PLAUR’s role in the establishment of an immunosuppressive microenvironment cannot be accounted for by M2 macrophages alone, and, perhaps in future work, we should focus more on other immune cell components, such as the MDSC population mentioned earlier.

TAMs, the predominant infiltrating immune cells in GBM tissue, constitute more than 30% of the total immune cell population [59]. Macrophages can directly induce the MES phenotype transition of glioma cells via cytokine-mediated pathways, such as the secretion of oncostatin M (OSM) by macrophages, which activates OSMR on GBM cells and triggers the switch to the MES molecular subtype [29]. Jason et al. demonstrated that TAMs originating from MES gliomas express high levels of MARCO and that TAMs with elevated MARCO expression have an enhanced capacity to promote the MES phenotype [60]. Therefore, the phenotypic heterogeneity of gliomas may stem from the heterogeneity of TAMs. In our study, we elucidated the association between PLAUR and the MES phenotype and further investigated whether the differential expression of PLAUR in TAMs contributed to MES glioma’s phenotype acquisition through modulating cell-to-cell interactions. We applied NicheNet analysis to identify the 20 ligands with the highest regulatory activity of MES marker gene expression. The expression levels of these ligands were then compared between distinct TAM subgroups classified by their PLAUR expression levels. It was observed that TAM cells with high PLAUR expression exhibited increased levels of ICAM-1, OSM, and other molecules known to mediate the MES phenotype of glioma. Thus, PLAUR can modulate macrophage–glioma cell interactions. This hypothesis was validated by the analysis of sequencing data and the tumor spheroid invasion assay using glioma primary cells (Figure 8). However, this part of the research had some limitations that should be acknowledged. Firstly, although we used glioma primary cells for the experiment, we only evaluated the invasion ability as a measure of the MES phenotype. Radiotherapy resistance is also a crucial feature of the MES phenotype, and related experiments are warranted in future work. Secondly, the mechanism by which PLAUR regulates OSM and ICAM-1 expression remains unclear. Lastly, beyond the interaction mediated by secreted components, the physical direct interaction between macrophages and tumor cells was not considered in the current study. Future work should address these issues so that we can better understand how PLAUR participates in glioma’s acquisition of the MES phenotype.

## 5. Conclusions

In conclusion, our study identified PLAUR as a hub gene of the GBM microenvironment. Our study integrated the features of hypoxia and immune infiltration and demonstrated PLAUR’s close association with the MES phenotype of GBM. By using the bioinformatics analysis of bulk and single-cell sequencing data, we revealed that PLAUR was highly expressed in both GBM cells and TAMs and that its expression was correlated with MES-related gene signatures. Moreover, we confirmed that PLAUR regulated the MES phenotype of GBM through both cell-intrinsic and cell-extrinsic mechanisms involving the modulation of macrophage–glioma interactions. These findings highlight the role of PLAUR in GBM progression and suggest that PLAUR might be a promising target for GBM treatment.

## Data Availability

All data supporting this study are available from the corresponding author upon reasonable request.

## Supplementary Materials

The following supporting information can be downloaded at: https://www.mdpi.com/article/10.3390/cancers16040840/s1, Figure S1: Identification of the hypoxia-immunosuppression status in GBMs; Figure S2: The correlation between PLAUR expression and clinicopathological characteristics of GBMs; Figure S3: PLAUR is associated with M2-like macrophages of GBM; Figure S4: PLAUR regulates the mesenchymal phenotype and tumor growth of glioma; Figure S5: Single-cell sequencing analysis of the dataset GSE131928; Figure S6: The potential of PLAUR on regulating MES phenotype through TAMs-to-glioma interaction; Table S1: Clinical characteristics of glioma patient cohort and applications; Table S2: Information about qPCR primers and RNA interfering target sequences; Table S3: Information about antibodies and reagents used in IHC, IF, WB, and flow; Table S4: List of pan-cancer immune metagenes; Table S5: Prognosis-associated genes defined by univariate Cox analysis; Table S6: Lasso coefficient profiles of 5 prognostic genes; original images for blots: uncropped images with molecular weight marker for all WB bands reported in figures.
