# Supplementary material for: Identifying PLAUR as a Pivotal Gene of Tumor Microenvironment and Regulating Mesenchymal Phenotype of Glioblastoma
Source: Cancers (Basel). 2024 Feb 19;16(4):840. doi: 10.3390/cancers16040840 (PMC10887327; doi:10.3390/cancers16040840)
Supplement: Supplementary file 1 [file cancers-16-00840-s001.zip › supplemental figures.pdf]

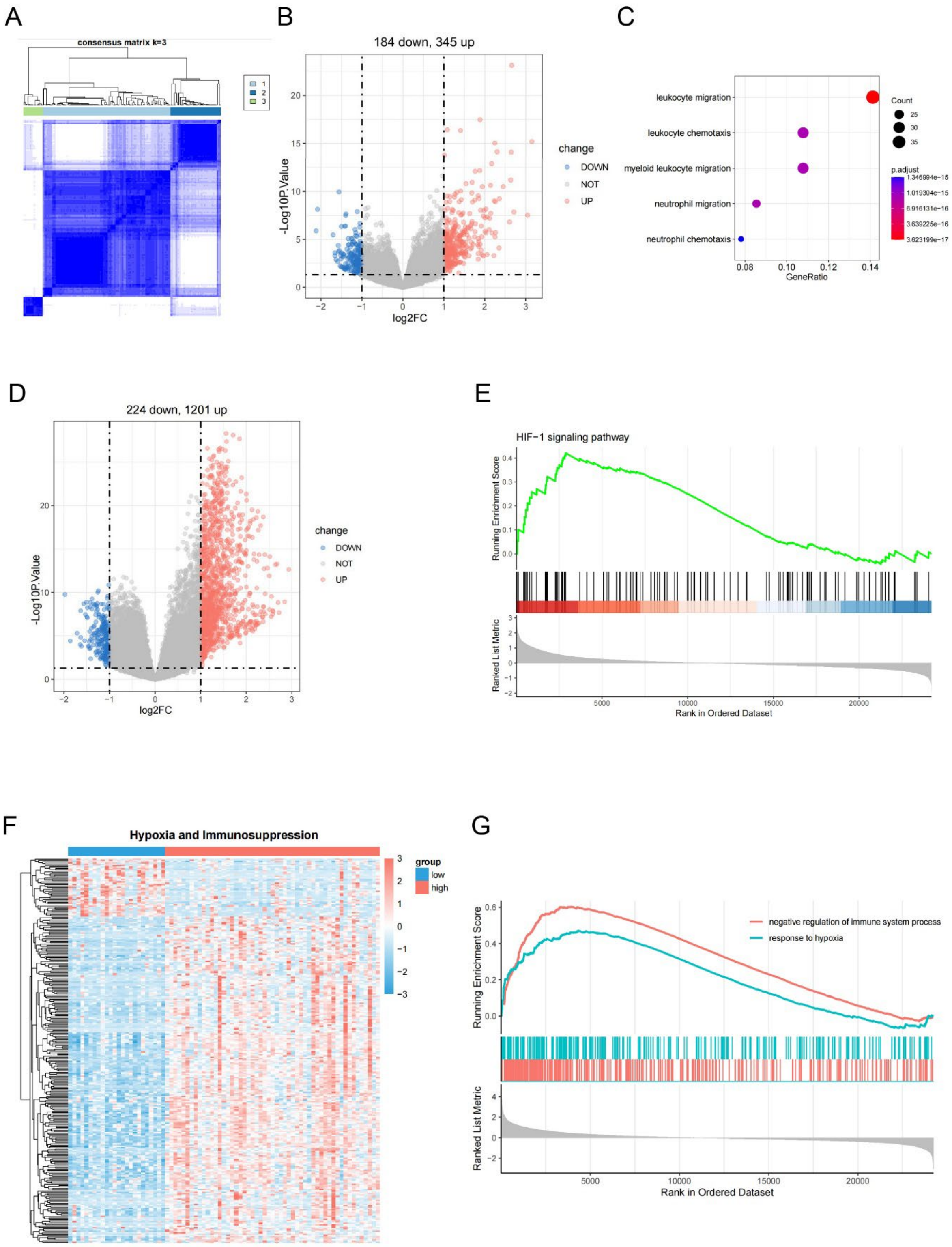

Figure S1. Identification of the hypoxia-immunosuppression status in GBMs. (A) Unsupervised hierarchical clustering of 152 TCGA-GBM samples based on the hypoxia score. (B) Volcano plot of DEG analysis between hypoxia high and low group. (C) GO enrichment analysis with hypoxia related DEGs. (E) GSEA analysis shows the positive enrichment of HIF-1 signaling in the IM high group. (F) Heatmap of differentially expressed genes in GBM samples grouped by HY-IM status. (G) GSEA analysis indicates the enrichment of immune and hypoxia functions with DEGs in F.

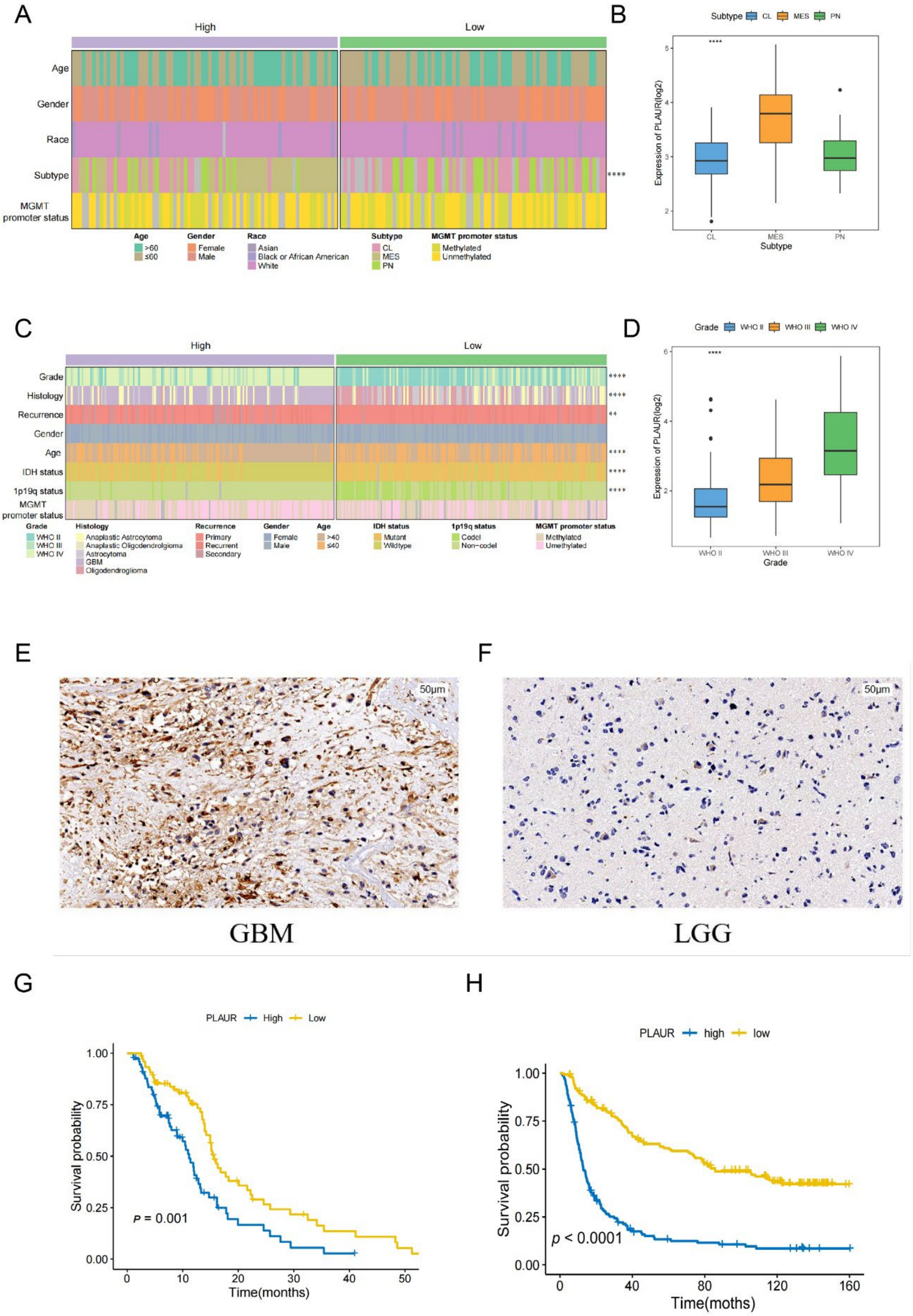

Figure S2. The correlation between PLAUR expression and clinicopathological characteristics of GBMs. (A) The plot shows the distribution pattern of clinical phenotypes in the PLAUR high and low groups in the TCGA-GBM cohort. Statistically significant phenotypes are labeled with asterisk. (B) Boxplot shows the highest expression of PLAUR in the MES subtype GBM. (C) The plot shows the distribution pattern of clinical phenotypes in the PLAUR high and low groups in the CGGA cohort. (D) Boxplot shows the expression of PLAUR in grade 2,3 and 4 gliomas. (E, F) Representative images of IHC staining for PLAUR in GBM and LGG tissue, respectively. Scale bar = 50μm. (G, H) Survival curve of patients in PLAUR high and low groups from TCGA-GBM and CGGA cohort, respectively. The significance was tested by the Log-rank test. *P <0.05, **P < 0.01, ***P < 0.001

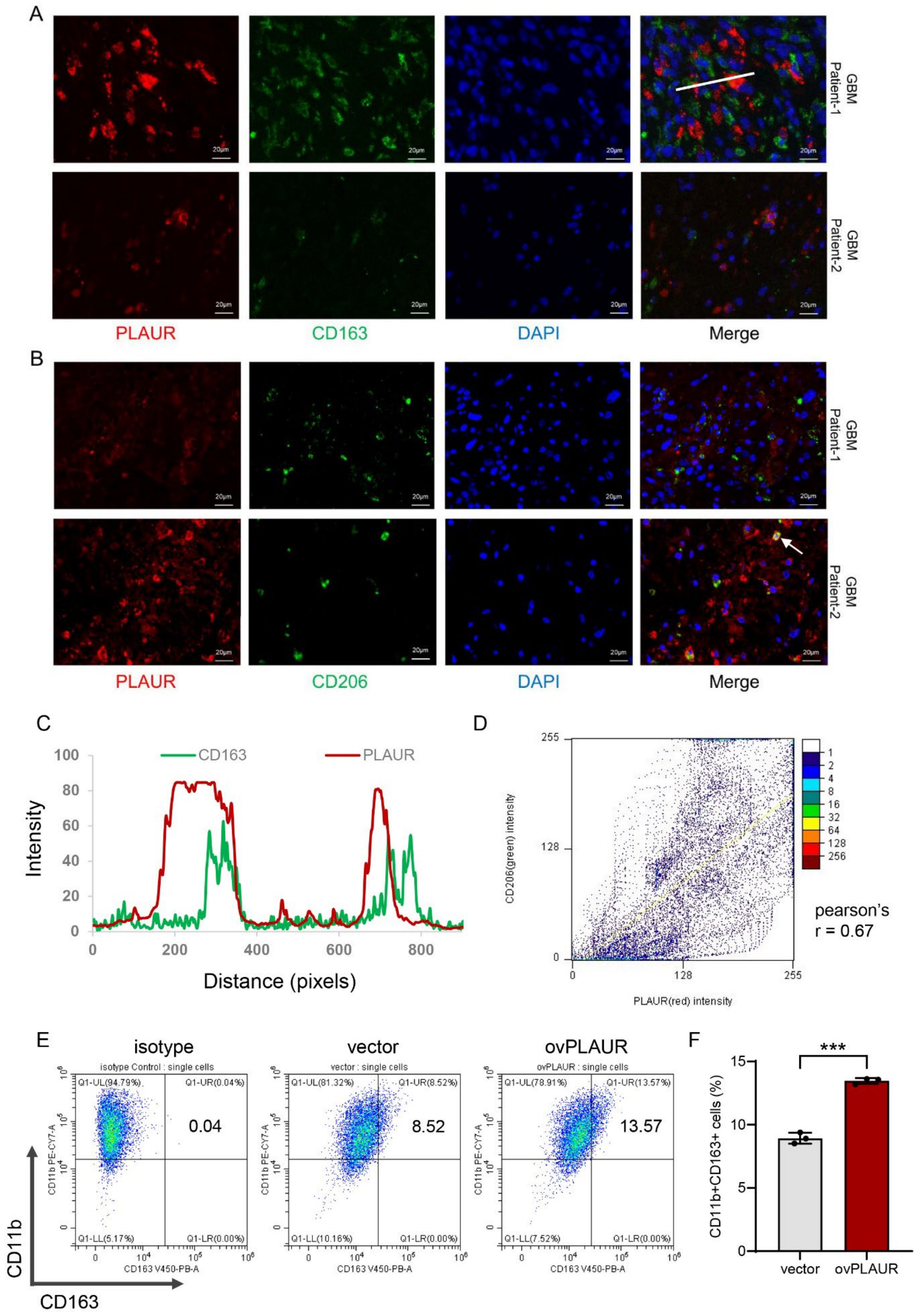

Figure S3. PLAUR is associated with M2-like macrophages of GBM. (A) Representative images of IF staining for PLUAR (red), CD163 (green), and nuclei were stained with DAPI (blue). The white line shows the ROI (Region of Interest) for the plot profile analysis. Scale bar = 20μm. (B) Representative images of IF staining for PLUAR (red), CD206 (green), and nuclei were stained with DAPI (blue). The white arrow indicates the cell for the colocalization analysis. Scale bar = 20μm. (C) The plot profile shows the adjacent distribution of PLAUR and CD163 in ROI. (D) The colocalization of PLAUR and CD206 was measured by the scatterJ plugin. (E) Flow cytometry of THP-1 macrophages with CD11b and CD163 antibodies. (F) The statistical analysis of CD11b+CD163+ cells in THP-1 macrophages transfected with PLAUR plasmid or vector control. **P* <0.05, ***P* < 0.01, ****P* < 0.001

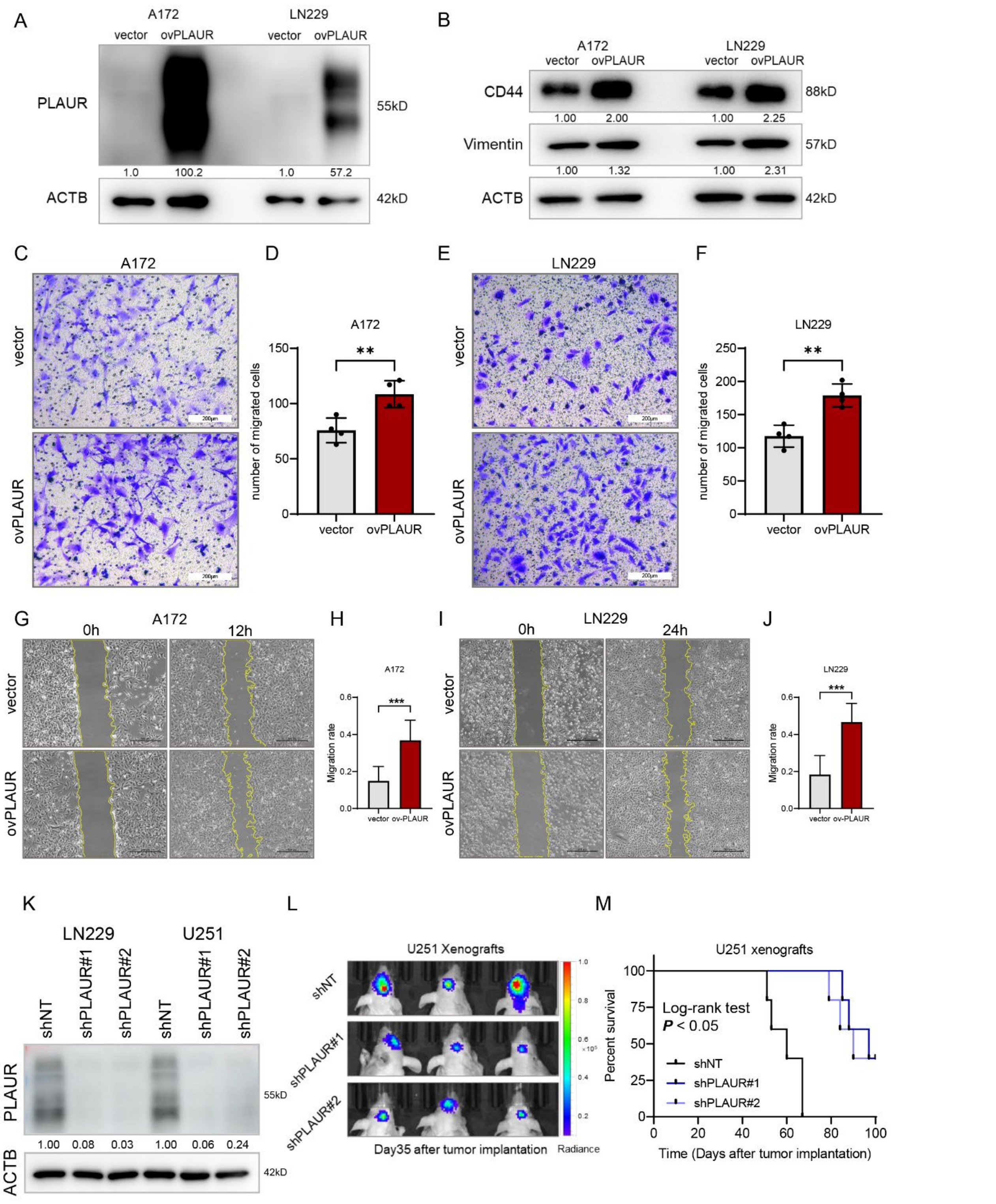

Figure S4. PLAUR regulates the mesenchymal phenotype and tumor growth of glioma. (A, B) Western blot analysis for PLAUR, CD44, and Vimentin expression with ACTB as endogenous control after PLAUR overexpression with plasmid in A172 and LN229. (C) Representative images of transwell assay for A172 with PLAUR overexpression. Scale bar = 200μm. (D) Statistical analysis for transwell assay of A172. (E) Representative images of transwell assay for LN229 with PLAUR overexpression. Scale bar = 200μm. (F) Statistical analysis for transwell assay of LN229. (G) Representative images of wound healing assay for A172 with PLAUR overexpression. Scale bar = 500μm. (H) Statistical analysis for wound healing assay of A172. (I) Representative images of wound healing assay for LN229 with PLAUR overexpression. Scale bar = 500μm. (J) Statistical analysis for wound healing assay of LN229. (K) Western blot for PLAUR expression in LN229 and U251 cell lines after knockdown mediated by lentiviral shRNA. (L) Bioluminescence imaging for xenografts constructed by shNT-U251 and shPLAUR-U251 at 35 days after tumor implantation. (M) The survival curve for mice in sh-NT and sh-PLAUR groups and statistical significance were tested by the Log-rank test. *P <0.05, **P < 0.01, ***P < 0.001

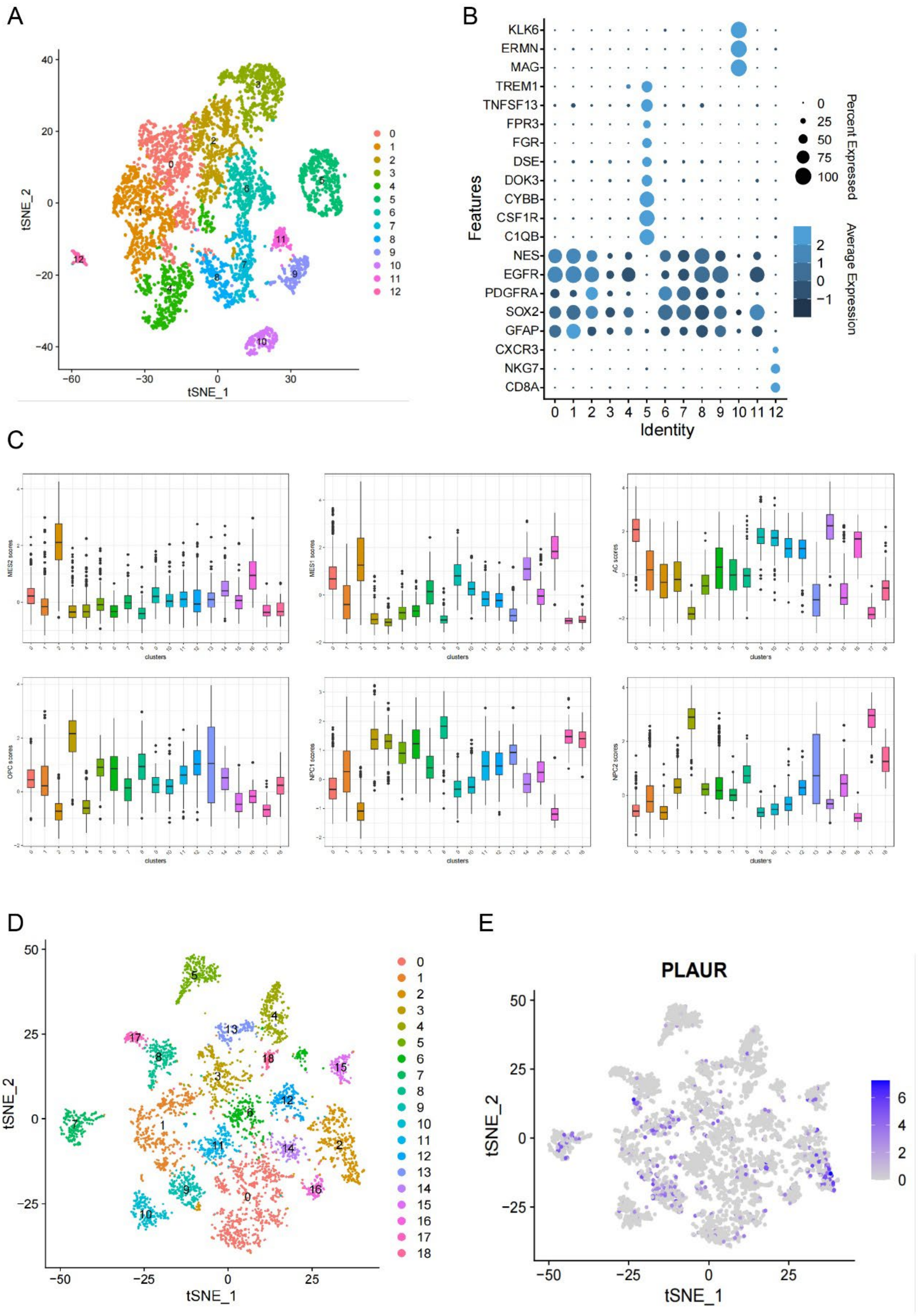

Figure S5. Single-cell sequencing analysis of the dataset GSE131928. (A) tSNE plot visualizes the dimensional reduction and cell clusters. (B) Dot plot of cell feature gene's expression in clusters. (C) Boxplots of the cellular state scoring for each cluster in malignancy (D) t-SNE plot of the clusters of malignant cells extracted from all cell types. (E) t-SNE plot of PLAUR expression level in the malignant clusters from D.

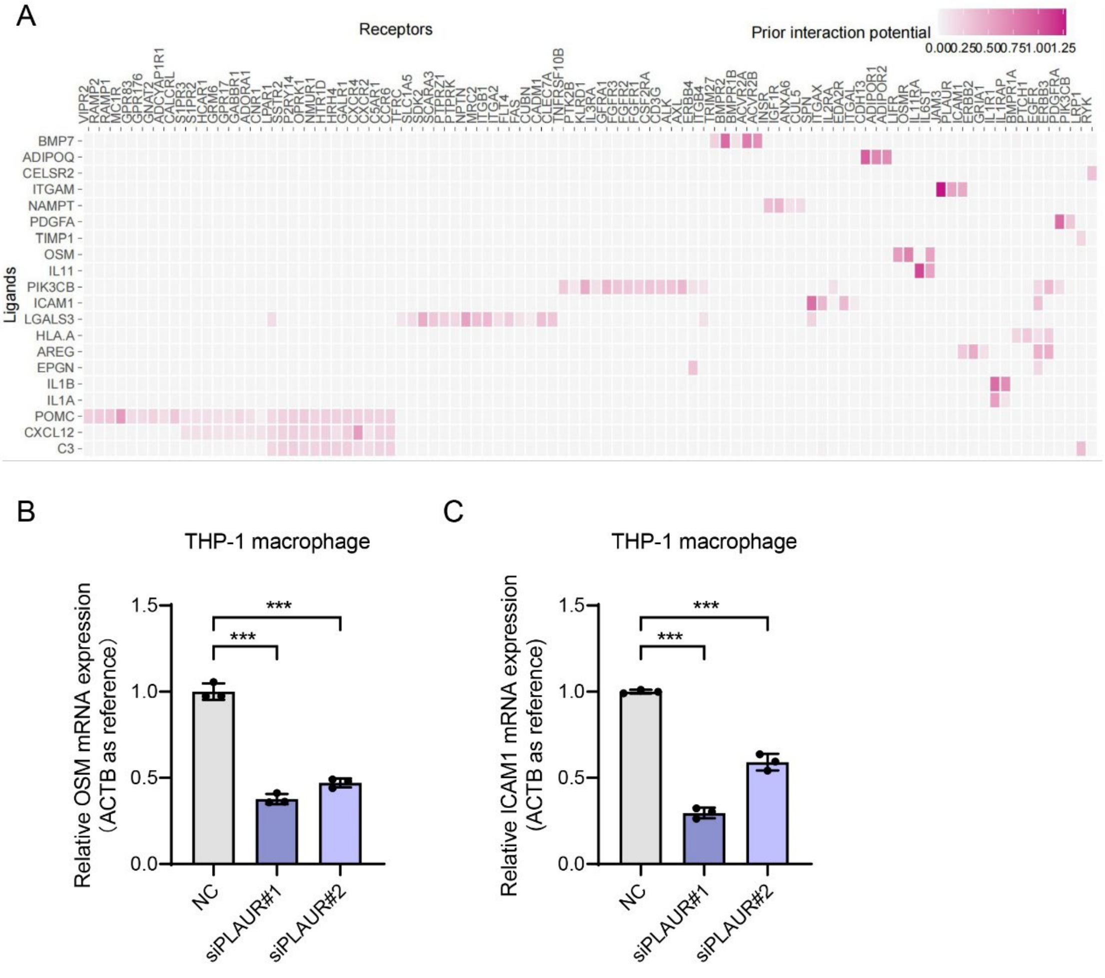

Figure S6. The potential of PLAUR on regulating MES phenotype through TAMs-to-glioma interaction. (A) Heatmap shows the interaction potential between ligands secreted by TAMs and receptors expressed on tumor cells. (B) Statistical analysis for OSM mRNA relative expression level of THP-1 derived macrophages with PLAUR silencing. (C) Statistical analysis for ICAM1 mRNA relative expression level of THP-1 derived macrophages with PLAUR silencing. *P <0.05, **P < 0.01, ***P < 0.001
