# Supplementary material for: Identifying PLAUR as a Pivotal Gene of Tumor Microenvironment and Regulating Mesenchymal Phenotype of Glioblastoma
Source: Cancers (Basel). 2024 Feb 19;16(4):840. doi: 10.3390/cancers16040840 (PMC10887327; doi:10.3390/cancers16040840)
Supplement: Supplementary file 1 [file cancers-16-00840-s001.zip › Table S1.pdf]

Table S1: Clinical characteristics of glioma patient's cohort and applications

The tumor samples were taken from patients operated on at The Second Affiliated Hospital of Zhejiang University School of Medicine for which enough tissue could be obtained for research. No further selection criteria were used.

Name	Sex	Location	Diagnosis	Primary or Recurrence	IDH	MGMT	Application
SZ21009	Male	right temporal	GBM(4)	Primary	WT	non-methylated	IHC,IF
SZ22002	Male	right frontal	GBM(4)	Primary	WT	non-methylated	IHC,IF
SZ22017	Male	left frontal and temporal	GBM(4)	Recurrence	WT	non-methylated	IHC
SZ22031	Female	right temporal and insular	Astrocytoma(3)	Primary	IDH1(R132H) MUT	/	IHC
GBM129	Female	left frontal	GBM(4)	Primary	WT	methylated	patient-derived glioblastoma cells
