# Supplementary material for: Identifying PLAUR as a Pivotal Gene of Tumor Microenvironment and Regulating Mesenchymal Phenotype of Glioblastoma
Source: Cancers (Basel). 2024 Feb 19;16(4):840. doi: 10.3390/cancers16040840 (PMC10887327; doi:10.3390/cancers16040840)
Supplement: Supplementary file 1 [file cancers-16-00840-s001.zip › Table S2.pdf]

Table S2: Information of qPCR primers and RNA interfering target sequences.

RNA interfering	Target Sequence	Source
homo-siPLAUR#1	5'-GCCGUUACCUCGAAUGCAU-3'	GenePharma,Shanghai,China
homo-siPLAUR#2	5'-CACTCTCCTCTGGACCTAAAC-3'	GenePharma,Shanghai,China
Lentiviral shPLAUR#1	5'-GCCGUUACCUCGAAUGCAU-3'	GeneChem,Shanghai,China
Lentiviral shPLAUR#2	5'-CACTCTCCTCTGGACCTAAAC-3'	GeneChem,Shanghai,China

qPCR primers	Sequence (5' to 3')
OSM Forward	TATAGGCAGCTGCTCGAAAG
OSM Reverse	CAGTTTAGGAACATCCAGGC
ICAM1 Forward	GACTAAGCCAAGAGGAAGGAGCAA
ICAM1 Reverse	TCAGCATACCCAATAGGCAGCAAG
ACTB Forward	CATGTACGTTGCTATCCAGGC
ACTB Reverse	CTCCTTAATGTCACGCACGAT
