# Supplementary material for: Identifying PLAUR as a Pivotal Gene of Tumor Microenvironment and Regulating Mesenchymal Phenotype of Glioblastoma
Source: Cancers (Basel). 2024 Feb 19;16(4):840. doi: 10.3390/cancers16040840 (PMC10887327; doi:10.3390/cancers16040840)
Supplement: Supplementary file 1 [file cancers-16-00840-s001.zip › Table S3.pdf]

Table S3: Information of antibodies and reagents used in IHC, IF, WB and Flow.

Antibody or reagent	Catalog Number	Dilution	Source
**IHC**			
uPAR Polyclonal antibody	10286-1-AP	1:100	Proteintech
**Flow**			
CD11b Monoclonal Antibody (M1/70), PE-Cyanine7	25-0112-82	1:200	Thermo Fisher Scientific
CD163 Monoclonal Antibody (MAC 2-158), eFluor™ 450	48-1637-42	1:200	Thermo Fisher Scientific
Mouse IgG1 kappa Isotype Control (P3.6.2.8.1), eFluor™ 450	48-4714-82	1:200	Thermo Fisher Scientific
**IF**			
uPAR (D7X2N) Rabbit mAb	12713	1:50	Cell Signaling Technology
CD163 Monoclonal antibody	68218-1-Ig	1:400	Proteintech
CD206 Monoclonal antibody	60143-1-Ig	1:400	Proteintech
Mounting Medium with DAPI	ab104139	/	abcam
CoraLite488-conjugated Goat Anti-Mouse IgG(H+L)	SA00013-1	1：400	Proteintech
CoraLite594 – conjugated Goat Anti-Mouse IgG(H+L)	SA00013-3	1：400	Proteintech
**WB**			
uPAR (D7X2N) Rabbit mAb	12713	1:1000	Cell Signaling Technology
CD44 (8E2) Mouse mAb	5640	1:1000	Cell Signaling Technology
Vimentin (D21H3) XP® Rabbit mAb	5741	1:3000	Cell Signaling Technology
Beta Actin Monoclonal antibody	66009-1-Ig	1:10000	Proteintech
HRP Goat Anti-Rabbit IgG (H+L)	AS014	1:5000	Abclonal Technology
HRP Goat Anti-Mouse IgG (H+L)	AS003	1:5000	Abclonal Technology
PageRuler Prestained Protein Ladder	26616	/	Thermo Fisher Scientific
