# Supplementary material for: Identifying PLAUR as a Pivotal Gene of Tumor Microenvironment and Regulating Mesenchymal Phenotype of Glioblastoma
Source: Cancers (Basel). 2024 Feb 19;16(4):840. doi: 10.3390/cancers16040840 (PMC10887327; doi:10.3390/cancers16040840)
Supplement: Supplementary file 1 [file cancers-16-00840-s001.zip › Table S4.pdf]

Table S4. List of Pancancer immune metagenes.

Cell type	Metagene
Activated B cell	ADAM28
	CD180
	CD79B
	BLK
	CD19
	MS4A1
	TNFRSF17
	IGHM
	GNG7
	MICAL3
	SPIB
	HLA-DOB
	IGKC
	PNOC
	FCRL2
	BACH2
	CR2
	TCL1A
	AKNA
	ARHGAP25
	CCL21
	CD27
	CD38
	CLEC17A
	CLEC9A
	CLECL1
Activated CD4 T cell	AIM2
	BIRC3
	BRIP1
	CCL20
	CCL4
	CCL5
	CCNB1
	CCR7
	DUSP2
	ESCO2
	ETS1
	EXO1
	EXOC6
	IARS
	ITK

	KIF11
	KNTC1
	NUF2
	PRC1
	PSAT1
	RGS1
	RTKN2
	SAMSN1
	SELL
	TRAT1
Activated CD8 T cell	ADRM1
	AHSA1
	C1GALT1C1
	CCT6B
	CD37
	CD3D
	CD3E
	CD3G
	CD69
	CD8A
	CETN3
	CSE1L
	GEMIN6
	GNLY
	GPT2
	GZMA
	GZMH
	GZMK
	IL2RB
	LCK
	MPZL1
	NKG7
	PIK3IP1
	PTRH2
	TIMM13
	ZAP70
Central memory CD4 T cell	ABHD3
	AHNAK
	ANXA2P2
	AQP3
	ATHL1
	BMI1
	BZW2

	CD63
	COL4A1
	CYLD
	ELMO2
	FYN
	GLIPR1
	GSS
	IFITM2
	ITGB1
	ITGB2
	KLF5
	LSP1
	NDUFB9
	PKM2
	SFXN3
	SIRPG
	SMAD4
	STX4
	TRADD
	VIM
	XRCC6
Central memory CD8 T cell	ACTN4
	ADAM12
	ADCY9
	F13A1
	FCER1G
	FCGR3B
	FGF7
	FKBP4
	GLUD1
	GM2A
	GUSB
	IL1RN
	NOL11
	NTRK1
	RARA
	RNF128
	SIGLEC1
	TNFRSF11A
	TOX4
	UBA52
	ULBP1
Effector memeory CD4 T cell	ATM

	CASP3
	CASQ1
	CD300E
	DARS
	DOCK9
	EXOSC9
	EZH2
	GDE1
	IL34
	NCOA4
	NEFL
	PDGFRL
	PTGS1
	REPS1
	SCG2
	SDPR
	SIGLEC14
	SIGLEC6
	TAL1
	TFEC
	TIPIN
	TPK1
	UQCRB
	USP9Y
	WIPF1
	ZCRB1
Effector memeory CD8 T cell	ACAP1
	APOL3
	ARHGAP10
	ATP10D
	C3AR1
	CCR5
	CD160
	CD55
	CFLAR
	CMKLR1
	DAPP1
	FCRL6
	FLT3LG
	GZMM
	HAPLN3
	HLA-DMB
	HLA-DPA1

	HLA-DPB1
	IFI16
	LIME1
	LTK
	NFKBIA
	SETD7
	SIK1
	TRIB2
Gamma delta T cell	ACP5
	AQP9
	BTN3A2
	C1orf54
	CARD8
	CCL18
	CD209
	CD33
	CD36
	CDK5
	IL10RB
	KLRF1
	LGALS1
	MAPK7
	KLHL7
	KRT80
	LAMC1
	LCORL
	LMNB1
	MEIS3P1
	MPL
	FABP1
	FABP5
	FADD
	MFAP3L
	MINPP1
	RPS24
	RPS7
	RPS9
	DBNL
	CCL13
Immature B cell	CD22
	CYBB
	FAM129C
	FCRL1

	FCRL3
	FCRL5
	FCRLA
	HDAC9
	HLA-DQA1
	HVCN1
	KIAA0226
	NCF1
	NCF1B
	P2RY10
	SP100
	TXNIP
	STAP1
	TAGAP
	ZCCHC2
Memory B cell	AICDA
	CCNA2
	CDKN3
	CLCN5
	ENPP1
	FCER1A
	FCRL4
	MYC
	RUNX2
	SORL1
	SOX5
	STAT5A
	STAT5B
	TLR9
Regulatory T cell	CCL3L1
	CD72
	CLEC5A
	FOXP3
	ITGA4
	L1CAM
	LIPA
	LRP1
	LRRC42
	MARCO
	MMP12
	MNDA
	MRC1
	MS4A6A

	PELO
	PLEK
	PRSS23
	PTGIR
	ST8SIA4
	STAB1
T follicular helper cell	B3GAT1
	CDK5R1
	PDCD1
	BCL6
	CD200
	CD83
	CD84
	FGF2
	GPR18
	CEBPA
	CECR1
	CLEC10A
	CLEC4A
	CSF1R
	CTSS
	DMN
	DPP4
	LRRC32
	MC5R
	MICA
	NCAM1
	NCR2
	NRP1
	PDCD1LG2
	PDCD6
	PRDX1
	RAE1
	RAET1E
	SIGLEC7
	SIGLEC9
	TYRO3
	CHST12
	CLIC3
	IVNS1ABP
	KIR2DL2
	LGMN
Type 1 T helper cell	CD70

	TBX21
	ADAM8
	AHCYL2
	ALCAM
	B3GALNT1
	BBS12
	BST1
	CD151
	CD47
	CD48
	CD52
	CD53
	CD59
	CD6
	CD68
	CD7
	CD96
	CFHR3
	CHRM3
	CLEC7A
	COL23A1
	COL4A4
	COL5A3
	DAB1
	DLEU7
	DOC2B
	EMP1
	F12
	FURIN
	GAB3
	GATM
	GFPT2
	GPR25
	GREM2
	HAVCR1
	HSD11B1
	HUNK
	IGF2
	RCSD1
	RYR1
	SAV1
	SELE
	SELP

	SH3KBP1
	SIT1
	SLC35B3
	SIGLEC10
	SKAP1
	THUMPD2
	TIGIT
	ZEB2
	ENC1
	FAM134B
	FBXO30
	FCGR2C
	STAC
	LTC4S
	MAN1B1
	MDH1
	MMD
	RGS16
	IL12A
	P2RX5
	CD97
	ITGB4
	ICAM3
	METRNL
	TNFRSF1A
	IRF1
	HTR2B
	CALD1
	MOCOS
	TRAF3IP2
	TLR8
	TRAF1
	DUSP14
Type 17 T helper cell	IL17A
	IL17RA
	C2CD4A
	C2CD4B
	CA2
	CCDC65
	CEACAM3
	IL17C
	IL17F
	IL17RC

	IL17RE
	IL23A
	ILDR1
	LONRF3
	SH2D6
	TNIP2
	ABCA1
	ABCB1
	ADAMTS12
	ANK1
	ANKRD22
	B3GALT2
	CAMTA1
	CCR9
	CD40
	GPR44
	IFT80
Type 2 T helper cell	ASB2
	CSRP2
	DAPK1
	DLC1
	DNAJC12
	DUSP6
	GNAI1
	LAMP3
	NRP2
	OSBPL1A
	PDE4B
	PHLDA1
	PLA2G4A
	RAB27B
	RBMS3
	RNF125
	TMPRSS3
	GATA3
	BIRC5
	CDC25C
	CDC7
	CENPF
	CXCR6
	DHFR
	EVI5
	GSTA4

	HELLS
	IL26
	LAIR2
Activated dendritic cell	ABCD1
	C1QC
	CAPG
	CCL3L3
	CD207
	CD302
	ATP5B
	ATP5L
	ATP6V1A
	BCL2L1
	C1QB
	SNURF
	SPCS3
	CCNA1
	CEACAM8
	NOS2
	SRA1
	TNFRSF6B
	TREM1
	TREML1
	RHOA
	SLC25A37
	TNFSF14
	TREML4
	VNN2
	XPO6
	CLEC4C
	TNFAIP2
	UBD
	ACTR3
	RAB1A
	SLA
	HLA-DQA2
	SIGLEC5
	SLAMF9
CD56bright natural killer cell	ABAT
	C11orf75
	C5orf15
	CDHR1
	DCAF12

	DYNLL1
	GPR137B
	HCP5
	HDGFRP2
	KRT86
	MLST8
	ELMOD3
	ENTPD5
	FAM119A
	FAM179A
	CLIC2
	COX7A2L
	CREB3L4
	CSF1
	CSNK2A2
	CSTA
	CSTB
	CTPS
	CTSD
	FST
	GATA2
	GMPR
	HDC
	HEY1
	HOXA1
	HS2ST1
	HS3ST1
	BCL11B
	CDH3
	MYL6B
	NAA16
	ClQA
	ClQB
	CYP27B1
	EIF3M
CD56dim natural killer cell	CYP27A1
	DDX55
	DYRK2
	RPL37A
	NOTCH3
	AKR7A3
	GPRC5C
	GRIN1

	HLA-E
	PORCN
	PSMC4
	UPP1
	IL21R
	KIR2DS1
	KIR2DS2
	KIR2DS5
Eosinophil	GIPR
	KRT18P50
	LRMP
	FOSB
	RRP12
	GPR183
	NR4A3
	ST3GAL6
	DEPDC5
	PDE6C
	PKD2L2
	GPR65
	IL5RA
	P2RY14
	DACH1
	DAPK2
	EMR3
Immature dendritic cell	ACADM
	AHCYL1
	ALDH1A2
	ALDH3A2
	ALDH9A1
	ALOX15
	AMT
	ARL1
	ATIC
	ATP5A1
	CAPZA1
	LILRA5
	RDX
	RRAGD
	TACSTD2
	INPP5F
	RAB38
	PLAU

	CSF3R
	SLC18A2
	AMPD2
	CLTB
	C1orf162
Macrophage	AIF1
	CCL1
	CCL14
	CCL23
	CCL26
	CD300LB
	CNR1
	CNR2
	EIF1
	EIF4A1
	FPR1
	FPR2
	FRAT2
	GPR27
	GPR77
	RNASE2
	MS4A2
	BASP1
	IGSF6
	HK3
	VNN1
	FES
	NPL
	FZD2
	FAM198B
	HNMT
	SLC15A3
	CD4
	TXNDC3
	FRMD4A
	CRYBB1
	HRH1
	WNT5B
Mast cell	ADAMTS3
	CPA3
	CMA1
	CTSG
	ARHGAP15

	CPM
	FCN1
	FTL
	HSPA6
	ITGA9
	RNASE3
	S100A4
	SIGLEC8
	SLC6A4
	PTGS2
	EGR3
	PILRA
MDSC	CCR2
	CD14
	CD2
	CD86
	CXCR4
	FCGR2A
	FCGR2B
	FCGR3A
	FERMT3
	GPSM3
	IL18BP
	IL4R
	ITGAL
	ITGAM
	PARVG
	PSAP
	PTGER2
	PTGES2
	S100A8
	S100A9
Monocyte	ASGR2
	CFP
	ASGR1
	CD1D
	UPK3A
	ACTG1
	ANXA5
	ATP6V1B2
	CFL1
	DAZAP2
	CTBS

	EMR4P
	HIVEP2
	MARCKSL1
	MBP
	MMP15
	PNPLA6
	TMBIM6
	PQBP1
	TEX264
	IKZF1
Natural killer cell	AKT3
	AXL
	BST2
	CDH2
	CRTAM
	CSF2RA
	CTSZ
	CXCL1
	CYTH1
	DAXX
	DGKH
	DLL4
	DPYD
	ERBB3
	F11R
	FAM27A
	FAM49A
	FASLG
	FCGR1A
	FN1
	FSTL1
	FUCA1
	GBP3
	GLS2
	GRB2
	LST1
	BCL2
	CDC5L
	FGF18
	FUT5
	FZR1
	GAGE2
	IGFBP5

	KANK2
	LDB3
Natural killer T cell	BTN2A2
	CD101
	CD109
	CNPY3
	CNPY4
	CREB1
	CRTC2
	CRTC3
	CSF2
	KLRC1
	FUT4
	ICAM2
	IL32
	LAMP2
	LILRB5
	KLRG1
	HSPA4
	HSPB6
	ISM2
	ITIH2
	KDM4C
	KIR2DS4
	KIRREL3
	SDCBP
	NFATC2IP
	MICB
	KIR2DL1
	KIR2DL3
	KIR3DL1
	KIR3DL2
	NCR1
	FOSL1
	TSLP
	SLC7A7
	SPP1
	TREM2
	UBASH3A
	YBX2
	CCDC88A
	CLEC1A
	THBD

	PDPN
	VCAM1
	EMR1
Neutrophil	CREB5
	CDA
	CHST15
	S100A12
	APOBEC3A
	CASP5
	MMP25
	HAL
	C1orf183
	FFAR2
	MAK
	CXCR1
	STEAP4
	MGAM
	BTNL8
	CXCR2
	TNFRSF10C
	VNN3
Plasmacytoid dendritic cell	CBX6
	DAB2
	DDX17
	HIGD1A
	IDH3A
	IL3RA
	MAGED1
	NUCB2
	OFD1
	OGT
	PDIA4
	SERTAD2
	SIRPA
	TMED2
	ENG
	FCAR
	IGF1
	ITGA2B
	GABARAP
	GPX1
	KRT23
	PROK2

	RALB
	RETNLB
	RNF141
	SEC14L1
	SEPX1
	EMP3
	CD300LF
	ABTB1
	KLHL21
	PHRF1
