# Supplementary material for: Identifying PLAUR as a Pivotal Gene of Tumor Microenvironment and Regulating Mesenchymal Phenotype of Glioblastoma
Source: Cancers (Basel). 2024 Feb 19;16(4):840. doi: 10.3390/cancers16040840 (PMC10887327; doi:10.3390/cancers16040840)
Supplement: Supplementary file 1 [file cancers-16-00840-s001.zip › Table S5.pdf]

Table S5：Prognosis-associated genes defined by univariate Cox analysis

Gene Symbol	HR	HR.95L	HR.95H	pvalue
HSPA7	2.214	1.465	3.345	0
PLAUR	1.943	1.299	2.908	0.001
TNFSF14	2.004	1.338	3	0.001
FCGR2C	2.001	1.34	2.99	0.001
MT1H	1.942	1.303	2.892	0.001
PDZK1IP1	1.93	1.289	2.891	0.001
HK3	1.858	1.244	2.776	0.002
THBD	1.908	1.268	2.87	0.002
CHI3L2	1.858	1.241	2.781	0.003
VDR	1.796	1.201	2.686	0.004
GJB2	1.813	1.205	2.728	0.004
AQP9	1.789	1.195	2.677	0.005
STC1	1.774	1.185	2.655	0.005
HIF1A-AS3	1.753	1.172	2.621	0.006
CXCL5	1.754	1.176	2.616	0.006
ADAMTS14	1.751	1.174	2.614	0.006
SPAG4	1.72	1.149	2.576	0.008
TREML3P	1.721	1.151	2.574	0.008
LINC01614	1.763	1.161	2.676	0.008
SAA2	1.686	1.132	2.51	0.01
TREM1	1.681	1.119	2.526	0.012
LILRB3	1.668	1.117	2.491	0.012
TNFAIP2	1.662	1.119	2.468	0.012
CD300H	1.665	1.119	2.479	0.012
SCNN1B	1.672	1.122	2.492	0.012
VENTX	1.653	1.113	2.456	0.013
RETN	1.661	1.115	2.474	0.013
MARCO	1.657	1.113	2.468	0.013
FCGR2B	1.63	1.099	2.418	0.015
LOX	1.625	1.094	2.416	0.016
LILRA5	1.634	1.093	2.442	0.017
BDKRB2	1.62	1.084	2.423	0.019
CFI	1.607	1.081	2.387	0.019
CLEC5A	1.598	1.078	2.368	0.02
SLC11A1	1.59	1.074	2.352	0.02
C1R	1.599	1.078	2.373	0.02
IBSP	1.606	1.079	2.39	0.02
SAA1	1.602	1.071	2.397	0.022

BX640514.2	1.588	1.062	2.375	0.024
AC007877.1	1.576	1.055	2.356	0.026
SPP1	1.556	1.052	2.301	0.027
NAMPT	1.533	1.032	2.276	0.034
LUCAT1	1.534	1.03	2.285	0.035
NDRG1	1.525	1.029	2.26	0.035
RNASE2	1.533	1.029	2.283	0.036
BIRC3	1.521	1.025	2.258	0.037
S100A8	1.528	1.024	2.281	0.038
COL5A1	1.524	1.024	2.268	0.038
CSTA	1.513	1.022	2.241	0.039
SLC16A3	1.511	1.019	2.241	0.04
PELATON	1.506	1.018	2.227	0.04
CEACAM4	1.524	1.019	2.28	0.04
CD163	1.514	1.014	2.262	0.043
ADAM8	1.499	1.012	2.222	0.044
COL6A2	1.503	1.01	2.237	0.044
CHI3L1	1.492	1.007	2.21	0.046
CD300E	1.495	1.007	2.218	0.046
PODNL1	1.493	1.005	2.217	0.047
ICAM1	1.488	1.004	2.205	0.048
