# Supplementary material for: Identifying PLAUR as a Pivotal Gene of Tumor Microenvironment and Regulating Mesenchymal Phenotype of Glioblastoma
Source: Cancers (Basel). 2024 Feb 19;16(4):840. doi: 10.3390/cancers16040840 (PMC10887327; doi:10.3390/cancers16040840)
Supplement: Supplementary file 1 [file cancers-16-00840-s001.zip › Table S6.pdf]

Table S6：Lasso Coefficient Profiles of 5 Prognostic Genes.

Gene	PLAUR	HSPA7	MT1H	PODNL1	PDZK1IP1
Coefficient	0.19580	0.05812	0.00368	0.09486	0.00634
